# Identifying monthly rainfall erosivity patterns using hourly rainfall data across India

**DOI:** 10.1038/s41598-025-11992-x

**Published:** 2025-07-31

**Authors:** Subhankar Das, Manoj Kumar Jain, Karl Auerswald, Carlos Rogerio de Mello, Peter Molnar

**Affiliations:** 1 Department of Hydrology, Indian Institute of Technology Roorkee, Roorkee, India; 2 School of Life Sciences, Technical University of Munich, Freising, Germany; 3 Water Resources Department, Federal University of Lavras, Lavras, Brazil; 4 Department of Civil, Environmental and Geomatic Engineering, ETH Zurich, Zurich, Switzerland

**Keywords:** Rainfall erosivity, R-factor, USLE, RUSLE, Soil erosion, India, Climate sciences, Hydrology, Natural hazards

## Abstract

Rainfall erosivity is a key dynamic factor of water erosion estimation, with a significant spatial and temporal variation. This study presents a comprehensive analysis of the spatial patterns and monthly distribution of rainfall erosivity across India, using data from 261 hourly and 2,525 monthly rainfall stations covering the period from 1969 to 2021. In India, monthly rainfall erosivity and related attributes—such as the kinetic energy of erosive rainfall, the number of erosive events, and peak hourly rainfall intensity—have been systematically examined for the first time. Monthly erosivity estimates derived from hourly data were linked with monthly rainfall, enabling a simplified and efficient estimation approach. To predict monthly erosivity based on rainfall, temperature, and topographic variables, we developed and evaluated three modeling approaches: linear regression, a machine learning-based XGBoost model, and an ensemble model. XGBoost outperformed the others, achieving a median coefficient of determination (R^2^) of 0.97, while the ensemble model also performed well with a median R^2^ of 0.96. Additionally, a Geographically Weighted Regression (GWR) approach was applied for spatial interpolation, yielding accurate high-resolution erosivity maps with a median R^2^ of 0.90. The results also demonstrate that erosivity peaks during the summer monsoon months (June to September), with July exhibiting the highest value due to intense rainfall and high kinetic energy. Notably, the analysis revealed that nearly 32% of India experiences monthly erosivity exceeding 2,000 MJ mm ha^−1 ^ h^−1 ^ month^−1 ^ in July alone. In contrast, non-monsoon months showed considerably lower erosivity levels across most of the country. A statistically significant long-term increase was detected in January, with an average rise of +0.86 MJ mm ha^−1 ^ h^−1 ^ month^−1 ^ in total erosivity and + 0.1 mm h^−1 ^ in maximum 60-min rainfall intensity annually. While acknowledging certain limitations, this study provides valuable insights into erosive rainfall characteristics, enhances rain-driven erosion assessment, and supports the development of timely and location-specific soil conservation strategies across India.

## Introduction

Water erosion is one of the most significant forms of environmental degradation affecting sustainable economies, food production, and socio-economic development in developing countries^1,2^. The global water erosion assessment showed that developing countries with less effective erosion management are more susceptible to erosion than developed countries^2,3^. South Asian countries are among the most vulnerable regions, characterized by intense rainfall, a growing population, degradation of natural resources, and high rates of poverty and food insecurity^4–7^. 

Predicting rainfall-induced water erosion using the Universal Soil Loss Equation (USLE)^8^ and its revised version, i.e., Revised Universal Soil Loss Equation (RUSLE)^9^ and RUSLE2^10^, requires spatially distributed and temporally highly resolved rainfall data. However, the limited availability of such rainfall datasets hinders accurate erosion estimation in many parts of the globe^11–15^. Erosion estimation using the USLE and its revised versions also requires an intra-annual dataset to account for the seasonality of rainfall and vegetation dynamics^13,16^. Notably, the seasonal and monthly distribution of rainfall erosivity is crucial for understanding soil loss dynamics and estimating the cover management factor^9,17^.

Accurate estimation of rainfall erosivity requires high-resolution precipitation data at 1- to 60-min intervals, ideally spanning more than 20 years^18–22^. It has been recognized that the unavailability of such high-temporal resolution observed datasets has resulted in significant errors in erosivity estimation^23–26^. Highly resolved satellite and climate reanalysis datasets are a promising alternative for estimating erosivity. However, recent rainfall erosivity studies in India^18,27,28^, the USA^29^, Burkina Faso^30^ and China^31^ showed a significant bias in the satellite and reanalysis-based erosivity estimates. Furthermore, the global assessment of satellite and reanalysis-derived erosivity products also revealed that most datasets failed to estimate erosivity correctly^11,14,32^. Notably, a significant underestimation was observed in the satellite and reanalysis-derived rainfall erosivity in the tropics^32^. 

Numerous empirical erosivity equations based on annual and monthly rainfall datasets are available^17,17,33–41^. Among these, one of the well-known empirical equations is based on the Fournier and modified Fournier index methods for estimating erosivity^33,42^. These equations have in common that they apply only regionally and may change in time due to climate change^43^. Various empirical erosivity equations have been applied for regional-scale erosivity estimation^39,44–46^, with some used for global-scale erosion studies^47–51^. 

India’s first national-level rainfall erosivity equation was developed almost four decades ago using rainfall datasets from 45 stations in different climate zones^34^. This equation was further used to develop seasonal and annual iso-erodent maps for the country. A linear regression was proposed to estimate erosivity based on the average annual rainfall of 44 stations, excluding Mahabaleshwar, due to its exceptionally high rainfall values^34,52,53^. Additionally, an erosivity equation for the monsoon season (June—September) was developed from these 44 stations to estimate erosivity for 180 additional locations distributed across the country. Finally, seasonal and annual iso-erodent maps were prepared from the 225 stations^34,53^. Notably, in 2004, Babu et al^35^ refined the annual and seasonal iso-erodent map by analyzing observed rainfall data from 123 stations. They developed new equations for estimating annual and seasonal erosivity from annual and monthly rainfall. These equations were applied to rainfall data from over 500 rain gauge stations, resulting in a comprehensive erosivity dataset from 623 stations for the updated iso-erodent map. 

Tiwari et al.^46^ utilized 101 years of monthly rainfall data from 52 stations across India to estimate erosivity using a modified Fournier index-based equation^33,42^. Notably, the equation applied for erosivity estimation was not explicitly developed for Indian conditions. Majhi et al.^26^ pointed out that the equation is a modified version of the original erosivity equation by Arnoldus^33^ developed for Morocco. Eventually, the same equation or different versions have been used in numerous studies in India^54–59^. Furthermore, Chen et al.^24^ identified nearly 28 modified versions of Arnoldus’s^33^ equation being used globally. Chen et al.^24^ also highlighted that China and India are the top two countries utilizing such equations despite not being developed for these regions. Despite Chen et al.^24^ emphasizing the need to stop the misuse of such equations, their application continues in many regional studies^54,55^. Additionally, Majhi et al.^26^ revealed that more than ten different methods, some developed for other regions^36,60,61^, have been employed for erosivity estimation in India using monthly and annual datasets. However, very few regional studies have utilized high-temporal resolution (1– 60 min) rainfall datasets^12,17,62,63^, indicating a significant gap.

In summary, the unavailability of high-temporal resolution and spatially dense rainfall stations has led to unreliable and erroneous rainfall erosivity estimates in India, which further cascade into errors in erosion estimation, ranging from plot scale to national level studies^26^. Furthermore, the use of unreliable erosivity equations across the country has raised questions about the accuracy of erosion estimates. Given the high demand for accurate erosion assessment, rain-driven damage estimation, and water resource management, precise rainfall erosivity estimates and an understanding of the intra-annual variability of erosivity in India are crucial. Therefore, in this study, we leverage more than 30 years of long-term rainfall data with high-temporal resolution (60-min) from the India Meteorological Department (IMD) to understand the erosivity characteristics and achieve accurate erosivity estimation. Notably, such a long-term, high-temporal resolution observed rainfall dataset has not been utilized in previous studies in India. Moreover, by utilizing a large collection of hourly and monthly rainfall datasets, we aim to provide more reliable and useful rainfall erosivity distribution surfaces across the country. Advances have been made by combining machine learning techniques and an advanced Geographically Weighted Regression (GWR) interpolation scheme to prepare high-resolution monthly rainfall erosivity maps for India. Additionally, temporal trends and spatial relationships with geo-climatic variables influencing rainfall erosivity were analyzed on a monthly scale. Specifically, we aim to:

1. Identify the long-term spatial and intra-annual pattern of erosivity based on hourly resolved long-term rainfall data.
2. Prepare long-term, highly resolved monthly rainfall erosivity surfaces or maps for India.
3. Quantify the long-term trends or patterns in monthly erosivity and its attributes.

### Study area

**Fig. 1:**
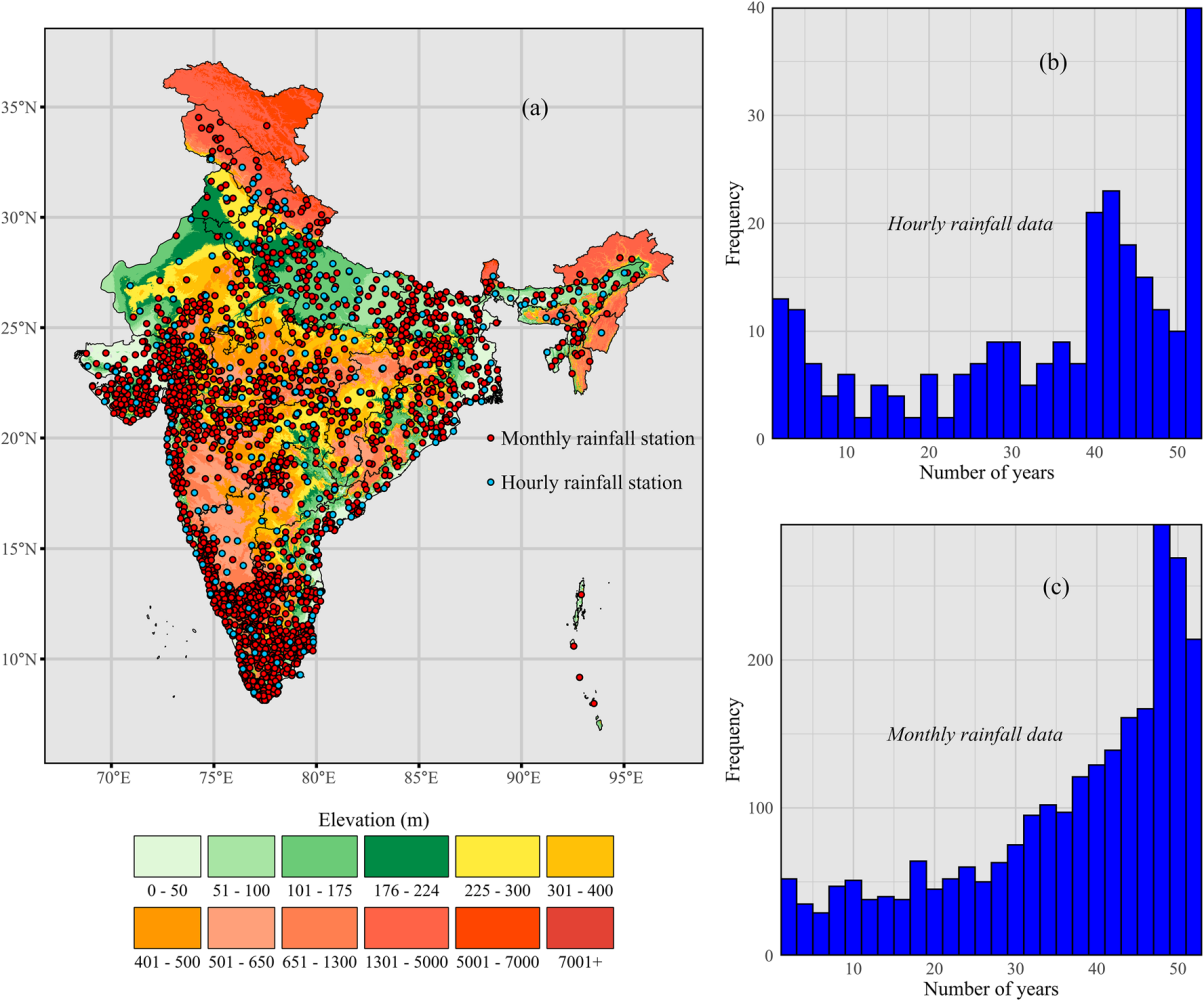
(**a**) Rainfall stations used for erosivity estimation from hourly and monthly rainfall data. (**b**) and (**c**) available temporal duration (years) of the hourly and monthly data. The map was generated using R version 4.4.3 with the *ggplot2*, *sf*, and *raster* packages.

India, located in the northern hemisphere, spans from approximately 8° 4′ N to 37° 6′ N latitudes and 68° 7′ E to 97° 25′ E longitudes, covering a vast area of approximately 3.287 million square kilometers, making it the seventh-largest country in the world (Fig. 1a). The country is bordered by China, Nepal, and Bhutan to the north, Pakistan to the west, and Bangladesh and Myanmar to the east. To the south, India is bounded by the Indian Ocean, with the Arabian Sea to its southwest and the Bay of Bengal to its southeast. A diverse geography, coupled with India’s vast size, contributes to a wide range of climatic conditions. They range from the Himalayan cold deserts in the north (Trans-Himalaya; see Supplementary Fig. 1) to the tropical rainforests in the south (in particular the Western Ghats) and the arid regions of the west (Thar desert) to the fertile plains of temperate climate in the north (Gangetic plains) and the east (West Bengal).

India’s climate is predominantly influenced by the monsoon seasons, with distinct wet and dry periods. The summer monsoon (June to September) brings heavy rainfall, particularly in the central highlands, northeast India, and the Western Ghats regions. At the same time, the western Himalayas and southern regions (Deccan Plateau, Western and Eastern Ghats) experience more variation in precipitation during pre-monsoon (March to May) and post-monsoon periods (October to November)^64^. India’s rainfall pattern shows marked spatial variability, with intense precipitation in the Western Ghats, northeast, and central regions, while arid zones like the Thar Desert receive as little as 100–500 mm annually. Notably, the northeastern state of Meghalaya hosts Mawsynram and Cherrapunji—among the wettest places on Earth—recording annual rainfall exceeding 10,000 mm^65^. This highly variable distribution is primarily shaped by orographic effects and the seasonal dynamics of the monsoon winds^66,67^.

With over 1.45 billion people, India is the most populous country in the world (https://www.worldometers.info/). The population is concentrated in urban centers and fertile agricultural regions, where large rural populations still rely on smallholder farming. The country’s dependence on agriculture, coupled with its vulnerability to climate change, poses a serious threat to food security and rural livelihoods (IPCC^5^). The increasing trend in rainfall events, particularly during the monsoon season, can lead to accelerated soil erosion, threatening agricultural productivity. Given that over half of India’s workforce is engaged in agriculture, such intense rainfall events have far-reaching socio-economic consequences, especially for marginal and smallholder farmers (FAO^68^).

### Datasets

This study incorporates an hourly rainfall dataset from 261 stations from 1969 to 2021, collected from the India Meteorological Department (IMD) (https://dsp.imdpune.gov.in/index.php). The start and end years of data records vary across stations. The collected dataset has an average coverage of nearly 35 years, with a median coverage of 41 years (Fig. 1b). Almost 80% of the stations have over 20 years of records, while approximately 90% of the stations have at least five years of hourly rainfall data. Additionally, a monthly rainfall dataset from 2,525 stations, spanning from 1969 to 2021, was collected from the IMD. The monthly dataset has an average duration of 37 years per station, with nearly 85% of the stations having more than 20 years of data, although some stations have less than 10 years of data (Fig. 1c).

Maximum and minimum temperature datasets from 1969 to 2021 were collected for 510 rainfall stations from the India Meteorological Department (IMD). However, temperature data for the remaining stations were unavailable, as many rain gauge stations are equipped only with non-recording rain gauges which do not capture temperature information. To fill this gap, the WorldClim (https://www.worldclim.org/)^69^ monthly dataset was used for these stations. Additionally, a high-resolution gap-filled Shuttle Radar Topography Mission (SRTM) elevation dataset, with a ~ 90-m spatial resolution, was obtained from the Consultative Group on International Agricultural Research—Consortium for Spatial Information (CGIAR-CSI) (https://srtm.csi.cgiar.org/). The same dataset was also used for slope estimation. We also obtained historical long-term climate variables—solar radiation (kJ m^−2^ day^−1 ^), wind speed (m s^−1 ^), and water vapor pressure (kPa) at a 30 arc-second (~ 1 km^2^) spatial resolution from WorldClim for the spatial mapping. Soil moisture (m^3^ m^−3^) and surface runoff (m) data were obtained from the ERA5-Land^70^ dataset ($0.1^\circ \times 0.1^\circ )$ of monthly averages (https://cds.climate.copernicus.eu/cdsapp). Soil type information (~ 250 m resolution) was sourced from the World Reference Base for Soil Resources 2006^71^, available at SoilGrids (https://soilgrids.org/). Land use data (~ 100 m resolution) were acquired from the Copernicus Land Monitoring Service (https://land.copernicus.eu/en). The ERA5-Land dataset was downscaled to the 30 arc-second resolution using a Random Forest-based downscaling method, as applied in our earlier study^6^. Soil and land use type datasets were upscaled using majority voting resampling^72^. The elevation (m) and slope (degree) estimated from the 90-m SRTM dataset were also converted to a 30 arc-second resolution using area-conservative regridding^32,73^. The coastline boundary was obtained from the Global Coast Line Dataset (GCL_FCS30)^74^, which was used to estimate the distance from the sea and is freely available at 10.5281/zenodo.13943679.

## Methods

### Erosivity estimation from hourly data

The rainfall erosivity, or R-factor, in the Revised Universal Soil Loss Equation (RUSLE), is the product of the total kinetic energy and the maximum 30-min rainfall intensity of erosive storms, typically derived from high-resolution pluviograph rainfall data^9,21,75,76^. However, due to the unavailability of pluviograph data, we used hourly rainfall datasets for rainfall erosivity estimation, as used in many earlier studies^12,29,77,78^. We identified erosive rain events based on the criteria provided by Renard et al.^9^, which are widely applied for such assessments. An erosive rain event is defined as one with a total precipitation of at least 12.7 mm, or a maximum 30-min intensity exceeding 12.7 mm h^−1 ^. The second criterion cannot be evaluated for hourly data. An erosive event is separated from the next rainfall event by at least a 6-h period of less than 1.27 mm rainfall. The kinetic energy for each unit of rainfall depth was calculated using the equation from RUSLE2^10,79^. The equation can be written as:

1 $$e = 0.29 \times \left[ {1 - 0.72 \times {\text{exp}}\left( { - 0.082 \times i} \right)} \right]$$

where $e$ is the rainfall kinetic energy per unit rainfall depth (MJ ha^−1 ^ mm^−1 ^), and $i$ is the rainfall intensity in mm h^−1 ^.

The total kinetic energy of erosive storms was estimated using Eq. 2.

2 $$E = \mathop \smallint \limits_{0}^{D} \left( {e \times \theta } \right) {\text{d}}t$$

where $E$ is the total kinetic energy (MJ ha^−1 ^) of the erosive storm. $\theta$ is the rainfall depth (mm) for each time increment ${\text{d}}t$ accumulated over rain duration *D*.

Equations 1 and 2 apply for increments of time with constant intensity, which can be assumed for short increments (minutes). For larger time increments like hours, intensity peaks are lost; the total kinetic energy and the maximum 30-min rainfall intensity become underestimated. This has to be corrected by correction factors^80,81^. This correction was applied when calculating the average monthly erosivity calculated from the event-based erosivity over the years of available data. The equation can be expressed as:

3 $$R_{month k} = \frac{1}{n}\mathop \sum \limits_{j = 1}^{m} K_{1} \times K_{2} \times \left( {EI_{60} } \right)_{j}$$

where, $R_{month k}$ is the long-term average monthly erosivity for month *k* in MJ mm ha^−1 ^ h^−1 ^ month^−1 ^, $n$ is the number of years of available hourly rainfall, $m$ is the total number of erosive storms that occurred in the month *k* in all *n* years. The $I_{60}$ is the maximum 60-min rainfall intensity of an erosive storm derived from the hourly rainfall data. Because the starting and ending years differ among stations, a long-term average monthly erosivity was estimated separately for each station and then averaged over the entire period. Furthermore, two conversion factors $K_{1}$ and $K_{2}$ convert 60-min erosivity to 1-min erosivity. The first conversion factor $K_{1}$ converts the rainfall erosivity from the 60-min rainfall resolution to the 15-min rainfall. A value of 1.678 was established by Das and Jain^12^ for this factor for Indian conditions. A value of 1.15 for factor $K_{2}$ was used based on Fischer et al.^80^ that converts the 15-min resolution to 1-min resolution. The product of $K_{1}$ taken from Das and Jain^12^ and $K_{2}$ from Fischer et al.^80^ yields 1.93, which is slightly lower than the conversion factor 2.05 found by Fischer et al.^80^ for Germany. In this study, we used a value of 2 for the product $K_{1}$ × $K_{2}$ (a trade-off of between 1.93 and 2.05). Thus, Eq. 3 simplifies to:

4 $$R_{month k} = \frac{1}{n}\mathop \sum \limits_{j = 1}^{m} 2 \times \left( {EI_{60} } \right)_{j}$$

### Monthly erosivity density

The concept of erosivity density (*ED*) was introduced in RUSLE2. ED quantifies how erosive rainfall is in a certain area or month. Small values of *ED* indicate gentle rainfall with little potential to cause erosion. In this case, erosivity is primarily driven by the total amount of rainfall. High values suggest that high-intensity rainstorms prevail^39,82,83^. *ED* is calculated as:

5 $$ED_{month} = \frac{{R_{month} }}{{P_{month} }}$$

where $ED_{month}$ is the monthly erosivity density in MJ mm ha^−1 ^ h^−1 ^ mm^−1 ^, $R_{month}$ is the long-term monthly rainfall erosivity in MJ mm ha^−1 ^ h^−1 ^ month^−1 ^, and $P_{month}$ is the long-term monthly precipitation in mm month^−1 ^.

### Erosivity estimation for stations with monthly climate data

Transfer functions are required to utilize the much higher number of stations with monthly data compared to the stations with hourly data. Traditionally, linear regressions are used for this task^39,84^. We used XGBoost (Extreme Gradient Boosting), a machine learning model^85,86^, to estimate monthly erosivity. For comparison, we also applied linear regression, given its widespread use in previous studies^34,35^. Additionally, we explored a hybrid approach (ensemble modelling^87^) that combines both XGBoost and linear regression models. All approaches utilized three spatially resolved climate variables—maximum temperature, minimum temperature, and monthly rainfall—along with two geographical covariates, elevation and slope, to estimate monthly erosivity (Eq. 6). Notably, all datasets were synchronized based on the availability of the rainfall data, ensuring consistent temporal coverage across all variables. These variables were selected based on an extensive review of previous research and due to the long-term availability of consistent datasets across India. Similar predictors have been used for the erosivity estimation in several earlier studies^15,39,88–90^.

6 $$R_{month} = f \left( {P_{month} ,Tmin_{month} , Tmax_{month} , Elevation, Slope} \right)$$

where $R_{month}$ is the monthly erosivity, $P_{month}$ is the monthly rainfall, $Tmin_{month}$ is the average minimum temperature, and $Tmax_{month}$ is the average maximum temperature of a particular month.

The function *f* can either be a linear regression model, similar to previous studies, for monthly erosivity estimation^39^:

7 $$R_{month} = \beta_{0} + \beta_{1} \times P_{month} + \beta_{2} \times Tmin_{month} + \ldots + \beta_{n} \times Slope$$

where $\beta_{0}$, $\beta_{1}$,…, $\beta_{n}$ are the regression coefficients that were obtained by using the *lm* function within the *Caret* package in R. Various combinations of independent parameters were tested to calibrate the most suitable multiple linear regression model based on the coefficient of determination for estimating monthly erosivity.

We also employed the XGBoost model to obtain *f*. Similar machine learning-based models have been found efficient in annual erosivity estimation^12,90–93^. XGBoost is known for its high efficiency and performance, especially with large datasets, due to parallel processing and tree-pruning techniques that reduce overfitting^94,95^. We used 70% of the erosivity dataset for calibration and 30% for the validation. We implemented tenfold cross-validation within the training dataset to prevent overfitting and ensure model generalizability. Each iteration used one of the 10 folds as a validation set for tuning the generalized model, while the remaining nine folds were used for training. The hyper-parameters of the XGBoost models were tuned to enhance model efficiency using a Bayesian optimization^96^. Specifically, hyper-parameters are external configurations or settings in machine learning that control the behavior of a learning algorithm and influence the model^97^. We used the *xgboost* package in R for modeling, and the hyper-parameters used in this study are provided in the Supplementary Table 1.

Additionally, an ensemble model was developed by combining linear regression and *XGBoost* models using the *SuperLearner* algorithm in R. This algorithm employs cross-validation to evaluate the performance of multiple machine learning models and determines the optimal weights for combining their predictions, resulting in an ensemble model. This approach was employed to assess whether combining the outputs of these two models leads to improved results. Similar methods have been applied in other research^98,99^. However, it should be noted that the model structures used in the individual models are not exactly the same as those in the combined model, as the *SuperLearner* algorithm operates with different hyper-parameters.

### Regionalisation of monthly erosivity

#### Geographically weighted principal component analysis (GWPCA)

The regionalization of station-based datasets was conducted using the Geographically Weighted Regression (GWR) and a Principal Component Analysis (PCA). Specifically, PCA was used to integrate efficient spatial covariates in the interpolation process. Traditional PCA often overlooks spatial heterogeneity among the factors^100^. However, recent advancements in integrating spatial information within PCA have revealed previously obscured details that may influence interpolation outcomes. The Geographically Weighted Principal Component Analysis (GWPCA)^101,102^, an advanced extension of traditional PCA, derives spatially varying principal components by computing the local variance–covariance matrix. The weighted matrix can be expressed as follows:

8 $$\sum \left( u \right) = X^{T} \times W\left( u \right) \times X$$

The $u$ represents the spatial location of the covariates, $X$ denotes the original co-variate matrix and $W\left( u \right)$ is the spatial location weight matrix. The weight matrix was computed using the Gaussian Kernel function.

The GW eigenvalues and eigenvectors were computed as:

9 $$L\left( u \right) \times V\left( u \right) \times L\left( u \right)^{T} = \sum \left( u \right)$$

where, $L\left( u \right)$ is the matrix of eigenvectors and $V\left( u \right)$ is the matrix of eigenvalues.

Finally, the GPWCA score matrix was calculated as:

10 $$S\left( u \right) = XL\left( u \right)$$

In this study, we utilized a 30-arc s spatial resolution dataset comprising rainfall (mm), elevation (m), slope (degree), solar radiation (kJ m^−2^ day^−1 ^), wind speed (m s^−1 ^), soil moisture (m^3^ m^−3^), surface runoff (m), soil types, land use types and water vapor pressure (kPa) as spatial covariates for the GWPCA. The cumulative proportion of variance explained by the principal components was used to determine the number of components to retain, with a threshold of at least 85% variance explained. The resulting GWPCA score matrix was then used as input covariates in the Geographically Weighted Regression (GWR) model.

#### Geographically weighted regression (GWR)

Spatial interpolation can be performed by methods that rely solely on the spatial structure of the data under focus (e.g., Inverse Distance Weighting (IDW), Kriging, and Spline Interpolation)^103^ or by approaches that additionally incorporate collocated covariates to enhance prediction accuracy^104^. The Geographically Weighted Regression (GWR)^105^ takes co-variables into consideration by calculating ordinary least squares regressions of the local relationships between these co-variables and an outcome of interest, giving greater weight to observations close to the location under focus than to observations further away^106^. This makes GWR particularly effective for analyzing spatial characteristics of rainfall, as the regression coefficients capture the regionally varying influence of co-variables^107^. As a result, GWR is superior for the interpolation of spatially varying variables compared to ordinary least square methods, inverse-distance weighting, or spline functions, and is similarly suitable as kriging^108,109^. The GWR relationship can be expressed as:

11 $$y \left( u \right) = \beta_{0} \left( u \right) + \mathop \sum \limits_{j = 1}^{n} \beta_{j} \left( u \right) \times x_{j} \left( u \right) + \varepsilon \left( u \right)$$

where $y \left( u \right)$ represents the dependent variables at location *u*, and $x_{j} \left( u \right)$ are the independent variables at that same location. The $\beta_{j} \left( u \right)$ represent the spatially varying parameters that need to be estimated for each location, and $\varepsilon \left( u \right)$ is the random error term.

Local regression models were constructed using observations at a given site $u$ and surrounding sites within a specific bandwidth by weighted least squares. The matrix can be expressed as:

12 $$\hat{\beta }\left( u \right) = \left( {X^{T} \times W\left( u \right) \times X} \right)^{ - 1} \times X^{T} \times W\left( u \right) \times y$$

where, $y$ is the $n \times 1$ vector of the dependent variable; $X$ is the matrix of the independent variables and $\hat{\beta }\left( u \right) = \left( {\widehat{{\beta_{0} }}\left( u \right) \ldots \widehat{{\beta_{n} }}\left( u \right)} \right)$ is the regression coefficient vector at site *u*. The weight matrix $W\left( u \right)$ applies geographical weights to each observation for the regression at point *i*. It is calculated with a kernel function based on the regression point *i* and *n* data points around it within a specific bandwidth. The Akaike information criterion was selected to determine the optimal bandwidth with a Gaussian kernel function automatically. The Gaussian kernel $w_{ij}$ can be written as:

13 $$w_{ij} = exp \left( { - \frac{1}{2}\left( {\frac{{d_{ij} }}{b}} \right)^{2} } \right)$$

where $w_{ij}$ is the weight between location $i$ and $j$. $d_{ij}$ is the distance between these locations, and $b$ is the bandwidth in the Gaussian kernel.

### Analyzing geo-climatic drivers of monthly erosivity

The spatial and temporal variation of rainfall erosivity is significantly influenced by geo-climatic variables (e.g. Mounirou et al^110^ in Africa, Chen et al.^111^ in China). Therefore, a geo-climatological assessment of monthly rainfall erosivity was conducted to understand its spatial and temporal variability across India. This analysis integrated key climatic drivers—rainfall, solar radiation, soil moisture, wind speed, water vapor pressure, and surface runoff—with geographical factors such as elevation, slope, distance from the sea, soil type, and land use. Monthly erosivity data from all stations were correlated with these eleven geo-climatic variables using the XGBoost model, as previously applied, to capture monthly variations and the underlying relationships among the variables.

To interpret the influence of each variable on the model output, we employed the SHAP (SHapley Additive exPlanations) approach using the *SHAPforxgboost* package in R. SHAP provides consistent and locally accurate explanations by decomposing model predictions into the additive contributions of individual input features^94,112^. This approach not only highlights the relative importance of each variable in explaining variations in rainfall erosivity but also reveals the direction (positive or negative) and nature of their influence across different months, offering a transparent and interpretable understanding of erosivity dynamics.

### Temporal trend analysis of erosivity and its attributions

The Modified Mann–Kendall test^113–115^ is an enhanced non-parametric method used for detecting trends in environmental and climate-related time series data, particularly when the assumption of data independence is compromised. In this study, the Modified M–K test, proposed by Hamed and Rao (1998)^115^, and implemented using the *modifiedmk* package in R, was applied to assess long-term trends in monthly rainfall erosivity attributes. This test is preferred over the standard Mann–Kendall test because it adjusts the variance based on the autocorrelation structure of the dataset, thereby reducing the risk of Type I errors and false trend detection. Autocorrelation is a common feature in hydroclimatic time series, especially for high-resolution monthly or daily data, and can inflate the likelihood of detecting spurious trends if uncorrected.

The null hypothesis of the Modified M–K test assumes that there is no trend (i.e., the data are independent and randomly ordered), while the alternative hypothesis suggests the presence of a trend. The magnitude of the trend in the time series was estimated using Sen’s slope estimator^116^. It provides a robust estimate of the median rate of change per unit time, with signs indicating the direction (positive or negative) of the trend.

Given that trend analysis was conducted on 96 combinations (12 months × 8 attributes), the risk of inflated Type I error due to multiple comparisons was addressed using the False Discovery Rate (FDR) correction to adjust the p-values. This approach helps control the expected proportion of false positives among the identified significant results, thereby enhancing the reliability and interpretability of the statistical inferences. By applying the FDR adjustment, we ensured that the detected trends reflect true underlying changes rather than random fluctuations.

### Metrics of error evaluation

We used three statistical metrics for accuracy assessment by applying the *hydroGOF* package in R: Percentage Error (PE), the coefficient of determination (R^2^), and the Root Mean Squared Error (RMSE). The description of these metrics can be found in previous studies^117,118^. They are calculated according to Eqs. 14 to 16. Additionally, we applied ANOVA (Analysis of Variance), a statistical method used to test whether the variation in the residuals can be explained by the variance in soil type, rainfall category, elevation, and land use category. A p-value of less than 0.05 was considered statistically significant, corresponding to a 95% confidence level in detecting an effect of the tested factor on the residual variability.

14 $$PE = \frac{{\left( {P_{i} - O_{i} } \right)}}{{O_{i} }} \times 100 \%$$

15 $$R^{2} = \left\{ {\frac{{\mathop \sum \nolimits_{i = 1}^{N} \left( {O_{i} - \overline{O}} \right)\left( {P_{i} - \overline{P}} \right)}}{{\sqrt {\mathop \sum \nolimits_{i = 1}^{N} \left( {O_{i} - \overline{O}} \right)^{2} } \sqrt {\mathop \sum \nolimits_{i = 1}^{N} \left( {P_{i} - \overline{P}} \right)^{2} } }}} \right\}^{2}$$

16 $$RMSE = \sqrt {\frac{{\mathop \sum \nolimits_{i = 1}^{N} \left( { O_{i} - P_{i} } \right)^{2} }}{N}}$$

where *O* is the observed value, *P* is the estimated value, $\overline{O}$ is the mean of observed values, $\overline{P}$ is the mean of estimated values, and *N* is the total number of values.

## Results

### Rainfall erosivity and attributes at stations with hourly rainfall data

**Fig. 2:**
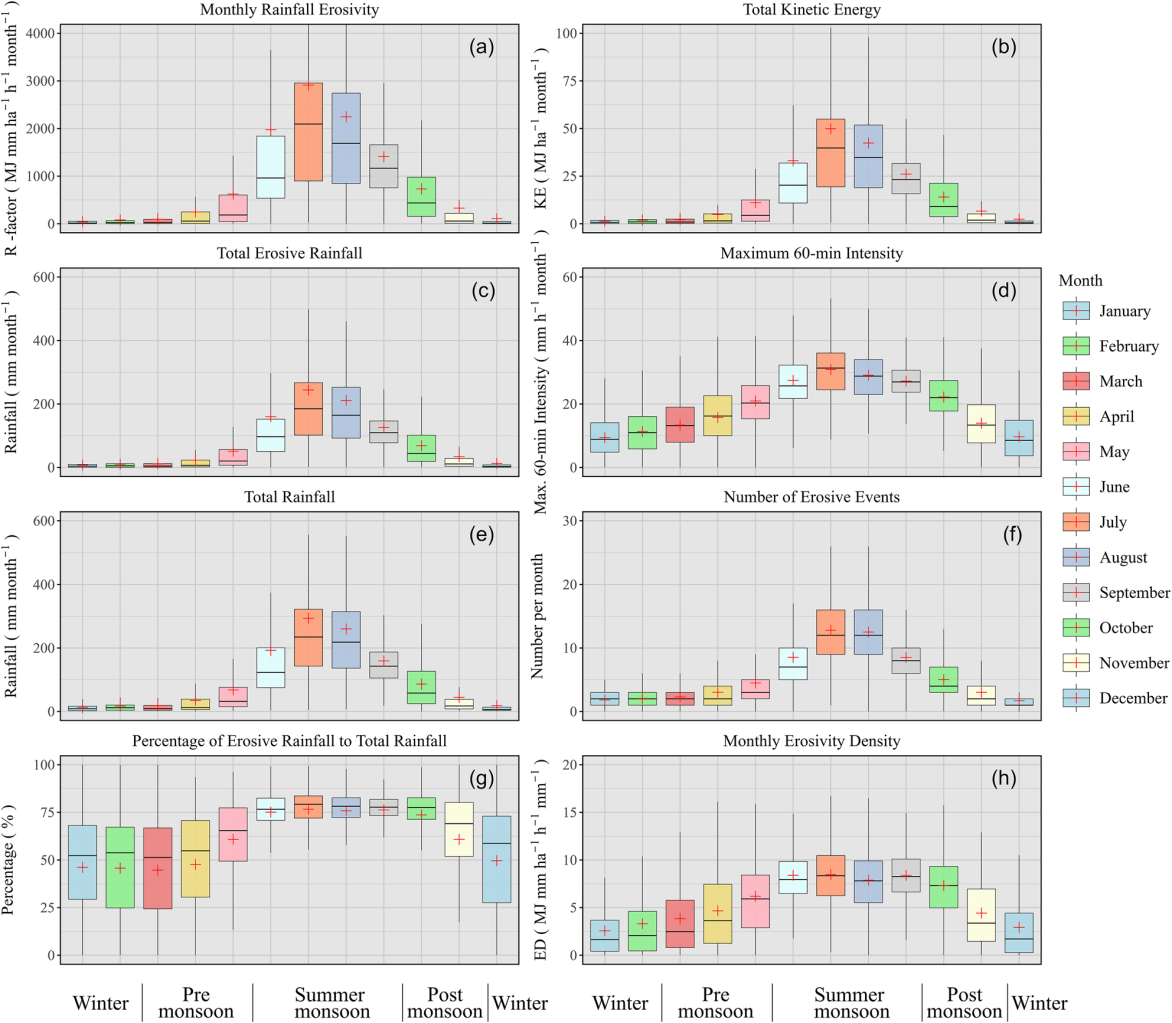
Monthly distribution of rainfall erosivity and its attributes for hourly rainfall stations in India, taking the long-term average of each station. The central line of the box plots indicates the median, the edges of the box represent the interquartile range, and the whiskers show the range within 1.5 times the interquartile range. The ‘ + ’ sign within the box indicates the mean value. For the minimum and maximum values, see Table 2 in the Supplementary. The plots were generated using R version 4.4.3 with the package *ggplot2*.

The long-term mean rain erosivity at stations with hourly rainfall data exhibited significant spatial and temporal variation (Fig. 2). Monthly erosivity values exceeded 20,000 MJ mm ha^−1 ^ h^−1 ^ month^−1 ^ at certain stations, while other stations recorded zero erosivity within the same months (for detailed minimum and maximum values, refer to Supplementary Table 2). Temporal variation was nearly as pronounced as spatial variation, with values approaching zero at most stations during the winter months (December and January).

When focusing on the majority of stations and disregarding the spatial pattern, three distinct seasonal patterns emerged for rain erosivity and its related attributes:

1. A marked peak in July and August was observed for rain erosivity (Fig. 2a), total kinetic energy (Fig. 2b), total erosive rainfall (Fig. 2c), total rainfall (Fig. 2e), and the number of erosive events (Fig. 2f). The two months preceding and following this peak showed either ascending or descending trends, while the remaining six months of the year exhibited very low values. This group of attributes, including rain erosivity, appeared to be primarily governed by total rainfall, which predominantly occurred in events large enough to be classified as erosive. Remarkably, the number of erosive events was larger than 15 for many stations between June and August, meaning that an erosive event occurred at least every second day. This was true for July and August for one-quarter of all stations. Notably, storm intensity was significantly high during these months.
2. A more gradual inter-month variation, also peaking in July, was found for the maximum 60-min intensity (Fig. 2d) and monthly erosivity density (ED) (Fig. 2h). The differences between months in this group were significantly smaller compared to the first group.
3. The third group comprised only the percentage of erosive rainfall relative to total rainfall (Fig. 2g). This attribute also exhibited a gradual change between months, similar to the second group, though with different within-month variability. From June to October, approximately 75% of total rainfall was erosive at 50% of the stations, while in the other months, the interquartile range was notably large (~ 50%), indicating pronounced spatial variation. This spatial variability was further highlighted by the minimum and maximum values (Table 2 in the Supplementary). In almost all months, the values ranged from 0%—where no rainfall was erosive over the multi-year observation period at specific stations—to 100%, where all rainfall was erosive at other stations.

Overall, the values of rainfall erosivity and its attributes generally followed the seasonal cycle, with low values in winter, intermediate values during the pre-monsoon and post-monsoon periods, and highest values during the summer monsoon. However, the alignment of the monsoon season with rainfall and erosivity attributes was only approximate. The pre-monsoon season exhibited values nearly as low as those in winter. The summer monsoon displayed considerable variation, with a continuous and pronounced increase starting low in May and peaking in August. The post-monsoon season began with values comparable to those observed in June, during the middle of the monsoon, before rapidly decreasing until November, when values were similar to the low levels recorded in December and January.

### Validation of the rainfall erosivity model for utilizing monthly stations

A correlation analysis between monthly erosivity and independent climate parameters indicated a strong correlation to monthly precipitation, especially during the summer monsoon months (r > 0.70; see Supplementary Figs. 2, 3, 4, 5, 6, 7, 8, 9, 10, 11, 12 and 13). Monthly minimum temperature showed a moderate correlation (r ~ 0.6) during the post-monsoon months, while other parameters demonstrated weaker correlations. The XGBoost model outperformed linear regression by far in most metrics (Table 1); for variable importance plots, refer to Supplementary Figs. 14, 15 and 16. XGBoost consistently achieved a median percentage error within ± 10% across almost all months, with January being the only notable exception (− 11.7%). In contrast, the linear regression model exhibited substantially larger errors, ranging from significant underestimations in February (-33.6%) and March (− 18.4%) to overestimations in April (+ 14.1%). The R^2^ and RMSE values further confirmed that regional XGBoost models outperformed the globaly used linear regression models and are better suited for estimating erosivity at stations with only monthly data.

**Table 1:** Evaluation metrics for the linear regression, XGBoost, and ensemble models used in monthly rainfall erosivity estimation.

Months	XGBoost			Linear regression			Ensemble model		
	Median PE	R^2^	RMSE	Median PE	R^2^	RMSE	Median PE	R^2^	RMSE
January	− 11.7	0.92	10	**− **8.4	0.45	62	**− **6.5	0.83	30
February	**− **3.7	0.94	11	**− **33.6	0.37	108	+ 2.1	0.90	50
March	**− **3.3	0.97	9	**− **18.4	0.60	170	**− **5.6	0.87	64
April	**− **1	0.98	9	+ 14.1	0.59	251	**− **2.7	0.97	105
May	**− **2.7	0.98	20	+ 7.5	0.87	680	**− **6.1	0.98	208
June	**− **1	0.97	67	**− **8.0	0.79	2267	**− **10.6	0.98	540
July	**− **5.4	0.97	134	**− **12.6	0.89	2266	**− **6.6	0.99	613
August	**− **1.5	0.98	100	+ 0.4	0.88	997	**− **6.1	0.96	633
September	+ 1	0.92	318	**− **8.9	0.66	478	**− **2.5	0.95	420
October	+ 2.4	0.97	169	**− **7.7	0.82	429	**− **1	0.94	202
November	**− **5.5	0.98	99	+ 12.1	0.81	193	+ 1	0.96	133
December	+ 4.1	0.98	16	**− **0.3	0.91	128	**− **8.8	0.99	44

Metrics were derived by applying the transfer functions developed from the calibration dataset (*n* = 183 hourly stations) to an independent test dataset (*n* = 78 hourly stations). PE, Percentage Error (%); R^2^, Coefficient of determination; and RMSE, Root Mean Squared Error (MJ mm ha^−1^ h^−1^ month^−1^).

Additionally, an ensemble model combining XGBoost and linear regression yielded better performance than linear regression alone. Although the ensemble model exhibited slightly higher RMSE values than XGBoost during certain winter months, it consistently outperformed linear regression across all months. Notably, the ensemble approach led to a significant reduction in RMSE (ranges from 30 to 633 MJ mm ha^−1 ^ h^−1 ^ month^−1 ^) and an improvement in R^2^ (ranges from 0.83 to 0.99) during the monsoon months. The XGBoost model, trained individually for each month with a significant number of hyper-parameters, demonstrated the lowest RMSE across all models, highlighting its robustness and effectiveness. Although the R^2^ values of the ensemble model were a little higher for some months, XGBoost consistently delivered superior overall performance. Therefore, this study adopts the XGBoost model for monthly erosivity estimation due to its superior predictive performance.

### Validation of erosivity regionalisation

Initially, simple kriging interpolation of monthly rainfall erosivity was performed using only the spatial distribution of the dataset, without incorporating additional variables. This approach resulted in substantial interpolation errors, with low predictive performance (R^2^ < 0.8 for all months; Supplementary Table 3). To improve accuracy, we adopted a more advanced methodology combining Geographically Weighted Principal Component Analysis (GWPCA) with Geographically Weighted Regression (GWR). The performance metrics of the GWPCA-GWR interpolation of monthly rainfall erosivity revealed significant variation throughout the year (Table 2). The percentage error ranged from + 11.5% in January to − 18% in May, with the lowest error observed in September (− 2.5%). The R^2^ values fluctuated between 0.72 in February and 0.98 in October, indicating variability in the model’s predictive accuracy. Notably, a strong median R^2^ of 0.90 was observed across the months, reflecting overall good model performance. The Root Mean Squared Error (RMSE) also exhibited significant differences across months, with the highest value recorded in July (499 MJ mm ha^−1 ^ h^−1 ^ month^−1 ^) and the lowest in January (12 MJ mm ha^−1 ^ h^−1 ^ month^−1 ^). The high value in July is most likely due to the elevated rainfall erosivity across India during this month, as July typically experiences significant rainfall throughout the country. Conversely, the lower R^2^ values during the winter months, such as January and February, can be attributed to minimal or zero erosivity. The limited variability in erosivity values during these periods presents challenges for interpolation models, as it hinders their ability to accurately predict erosivity.

**Table 2:** Spatial interpolation error metrics for the monthly erosivity mapping using a combination of Geographically Weighted Principal Component Analysis (GWPCA)-Geographically Weighted Regression (GWR) from the calibration dataset (*n* = 1950 hourly and monthly stations) when applied to the test dataset (*n* = 836 hourly and monthly stations).

Months	Median PE	R^2^	RMSE
January	+ 11.5	0.73	12
February	− 3.0	0.72	28
March	**− **6.8	0.89	39
April	**− **12.9	0.89	88
May	**− **18	0.88	243
June	**− **6.9	0.88	263
July	**− **9.1	0.90	499
August	**− **4.4	0.93	482
September	**− **2.5	0.91	336
October	**− **4.2	0.98	171
November	**− **2.6	0.95	50
December	+ 4.9	0.92	23

PE, Percentage Error (%); R^2^, Coefficient of determination; and RMSE, Root Mean Squared Error (MJ mm ha^−1^ h^−1^ month^−1^).

### Regionalisation of annual and monthly erosivity

**Fig. 3:**
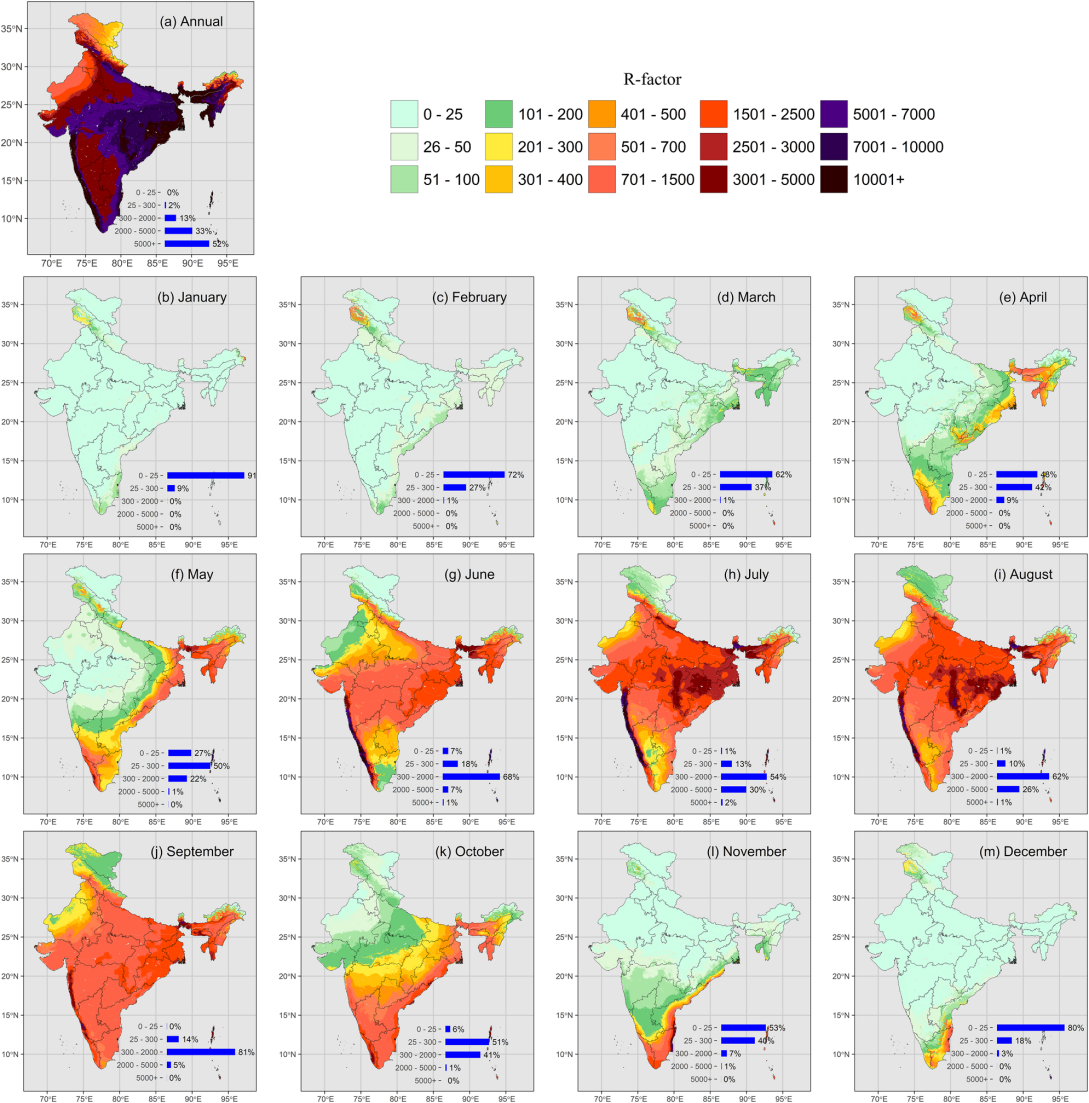
Estimated long-term average (1969—2021) of annual and monthly erosivity across India. Units differ by panel: a) MJ mm ha^−1 ^ h^−1 ^ year^−1 ^ for annual erosivity; b) to m) MJ mm ha^−1 ^ h^−1 ^ month^−1 ^ for the monthly erosivity. Rounded areal percentages are shown as bar plots at the bottom of each panel; minor rounding discrepancies may result in slight deviations. For prediction errors associated with the maps, refer to Table 2. The maps were generated using R version 4.4.3 with the packages *ggplot2*, *sf*, and *raster*.

Annual rainfall erosivity in India exhibited a highly variable spatial distribution (Fig. 3a), ranging from as low as 500 MJ mm ha^−1 ^ h^−1 ^ year^−1 ^ in narrow, rain-shadowed valleys of the Western Himalayas and the northeastern tip of the Eastern Himalayas to values exceeding 20,000 MJ mm ha^−1 ^ h^−1 ^ year^−1 ^ in regions such as the Western Ghats, the central highlands, and the southern slopes of the Khasi Hills. The spatial gradients of rain erosivity were particularly pronounced in the northeast, where the full range of values was observed within distances of ~ 200 km. A significant portion of India, including the semi-arid northwest (mean rainfall erosivity values for different climatic conditions can be found in Supplementary Tables 4 and 5), the Deccan Plateau, and the western Gangetic Plain, experiences annual erosivities between 2,000 and 5,000 MJ mm ha^−1 ^ h^−1 ^ year^−1 ^. In contrast, low erosivity values, ranging from 400 to 700 MJ mm ha^−1 ^ h^−1 ^ year^−1 ^, are characteristic of the Thar Desert and the Trans-Himalayan region.

The spatial variability of erosivity became even more pronounced when examined on a monthly basis, as the temporal distribution of rain erosivity differed significantly across regions, partially averaging out in the annual totals. During January and February, rain erosivity was minimal across most of India, with the exception of the Trans-Himalayas (Fig. 3b, c). More than 70% of the country exhibited erosivities below 25 MJ mm ha^−1 ^ h^−1 ^ month^−1 ^ in January and February. By late March, as the high-sun season progresses toward the Equator, increasing atmospheric instability and convective rainfall cause erosivity to rise, particularly along the eastern parts of India (Fig. 3d), a trend that intensifies in April and May (Fig. 3e, f). Erosivities already reached values of 2,000 MJ mm ha^−1 ^ h^−1 ^ month^−1 ^, and 2% of the country even exceeded this value in May. High erosivity values also emerged during this period in the Cardamom Hills, the Eastern Ghats, and the Northeast.

With the onset of the summer monsoon in early June, which advances from the south and reaches the northern regions, excluding the Thar Desert, by the end of the month, rain erosivity became uniformly high across most of India (Fig. 3g). Erosivity was within a range of 300 to 2,000 MJ mm ha^−1 ^ h^−1 ^ month^−1 ^ in two-thirds of India. By July, the monsoon—and the associated high erosivity—extends into the Thar Desert as well (Fig. 3h), a pattern that continues through August (Fig. 3i). This relatively uniform pattern persisted until September (Fig. 3j). During the peak of the monsoon season, from July to August, the increased monsoonal activity over the Arabian Sea significantly amplified erosivity in the Western Ghats, where orographic effects lead to spectacular rainfall as moist monsoon winds are blocked by the steep slopes. As a result, the Western Ghats experienced markedly higher erosivities during these months compared to other regions, even exceeding 5,000 MJ mm ha^−1 ^ h^−1 ^ month^−1 ^.

By early October, the winter monsoon begins, lasting until December. It brings rain to the southeastern Deccan Plateau and the Eastern Ghats. In October, high erosivity was observed along the entire eastern part of India, but by December, it retreated to the southeastern tip of India (Fig. 3k–m). Consequently, the southern tip of India experienced a prolonged erosion season, spanning from April to December. The Western Himalayas also exhibited a comparably even temporal distribution in rain erosivity. In contrast, the Western Ghats experienced extremely high erosivity concentrated within just three months—June, July, and August—while the Thar Desert had an even shorter erosion season, limited to July and August.

### Influence of geo-climatic variables on monthly erosivity

**Fig. 4:**
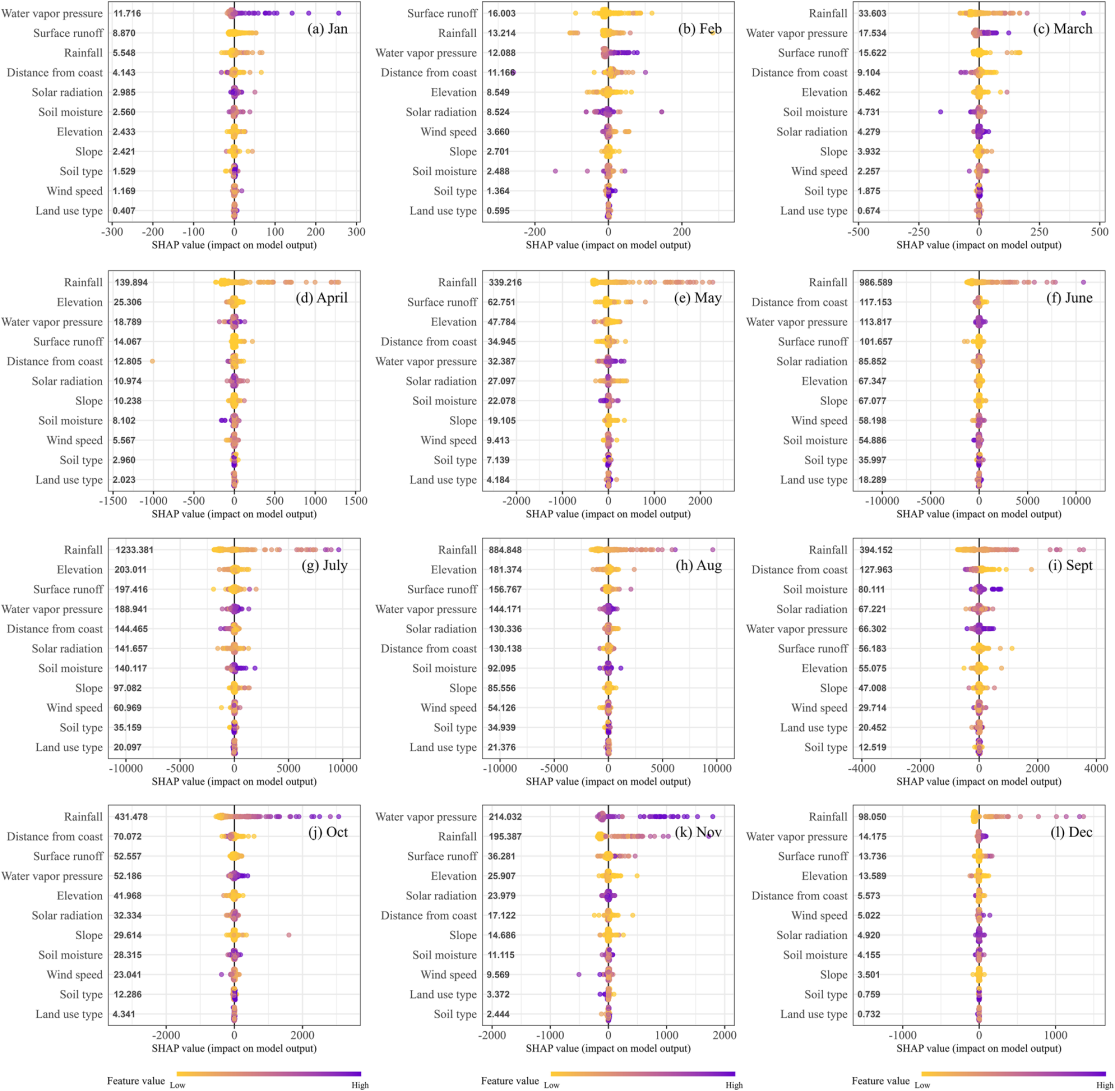
SHAP (SHapley Additive exPlanations) summary plot showing the importance of geo-climatic predictors contributing to the spatial variation of rainfall erosivity across India. The variables on the Y-axis are ranked by their importance, from high (top) to low (bottom), based on their average SHAP values. Each point represents a SHAP value for an individual observation, with color indicating the magnitude of the feature value—violet for high values and yellow for low values. The plot illustrates the contribution of each geo-climatic variable to rainfall erosivity, with wider distributions indicating greater variability in feature impact across the dataset. The plots were generated using R version 4.4.3 with the package *ggplot2*.

The SHAP (SHapley Additive exPlanations) analysis in Fig. 4 highlights the monthly contribution of geo-climatic variables to the estimation of rainfall erosivity across India. The results reveal notable seasonal variability in the influence of predictors, which reflects the dynamic nature of erosivity processes under varying climatic and geographic conditions. To better understand the role of individual variables throughout the year, we grouped the predictors based on the frequency of their relative importance across the months (details have been summarized in the Supplementary Table 6). The rankings were categorized as high, medium, or low importance using their SHAP value magnitudes and positions.

1. High Importance (frequently among the top 3 contributors): Rainfall consistently emerged as the dominant driver of erosivity across all months, with its influence peaking during the summer monsoon period (June to September) (Fig. 4f–i); July recorded the highest importance score, underscoring rainfall’s critical role during peak erosive months. Surface runoff also exhibited a strong influence, particularly in the pre-monsoon (Fig. 4c–e) and summer monsoon seasons, emphasizing its control over erosive energy and soil detachment. Additionally, water vapor pressure demonstrated significant importance, especially during the winter (Fig. 4a, b, and l) and post-monsoon (Fig. 4j and k) months, where it contributed notably to erosivity processes during drier periods.
2. Medium Importance (intermittent or moderate influence): Distance from the coast showed moderate importance during the pre-monsoon and winter seasons, likely due to its impact on regional moisture availability and atmospheric dynamics. Solar radiation contributed moderately during several months, particularly in transitional periods, reflecting its role in influencing storm intensity and evaporation. Elevation also demonstrated a moderate influence, affecting rainfall distribution and erosivity gradients across varying terrains, especially during the pre-monsoon and monsoon months.
3. Low Importance (consistently low SHAP rankings): Soil moisture generally ranked low in importance, with only occasional influence observed in specific months (e.g., September). Wind speed also played a minor role throughout the year, likely due to its indirect or limited connection to monthly-scale erosivity processes. Similarly, slope consistently showed minimal importance across all months, suggesting a secondary role in influencing erosivity at the national scale. Additionally, soil type and land use exhibited consistently low SHAP values, indicating that their contribution to monthly erosivity estimation was relatively minor across India.

### Long-term trends in monthly erosivity attributes

There were significant changes in 33 out of 96 cases (12 months × 8 attributes) (Table 3) based on the Modified Mann–Kendall test (see p-values in Supplementary Table 7). However, this number must be interpreted cautiously, as the probability of false positives (Type I errors) is high due to multiple testing. Notably, all erosivity-related attributes with significant changes showed increasing trends, except for the number of erosive events, which exhibited a decreasing trend in six out of 12 months, with only one month (August) showing a statistically significant decline.

**Table 3:** Sen’s slope estimates per year for monthly rainfall erosivity and its contributing components, derived from the spatial average of 261 hourly stations across India during the period 1969–2021.

Month	Total erosivity	Total kinetic energy	Total erosive rainfall	Maximum 60-min intensity	Total rainfall	Number of erosive events	Percentage of erosive rainfall to total rainfall	Monthly erosivity density
January	+ 0.86	**+ **0.02	**+ **0.10	**+ **0.10	**+ **0.10	**− **0.01	**+ **0.25	**+ **0.06
February	**+ **0.34	**+ **0.01	**+ **0.04	**+ **0.04	**+ **0.03	**− **0.01	**+ **0.21	**+ **0.02
March	**+ **0.98	**+ **0.02	**+ **0.07	**+ **0.03	**+ **0.09	**− **0.02	**+ **0.15	**+ **0.02
April	**+ **0.58	**+ **0.03	**+ **0.10	**+ **0.00	**+ **0.13	**− **0.02	**+ **0.05	0.00
May	**+ **3.18	**+ **0.03	**+ **0.10	**+ **0.06	**+ **0.13	0.00	**+ **0.06	**+ **0.03
June	**+ **0.15	**+ **0.01	**− **0.03	**+ **0.02	**− **0.01	0.00	**+ **0.00	0.00
July	**+ **7.40	**+ **0.11	**+ **0.44	**+ **0.03	**+ **0.33	**− **0.01	**+ **0.06	**+ **0.01
August	**− **0.34	**− **0.06	**− **0.39	**+ **0.02	**− **0.45	**− **0.03	**− **0.02	**+ **0.02
September	**+ **3.47	**+ **0.07	**+ **0.34	**+ **0.03	**+ **0.36	0.00	**+ **0.05	**+ **0.01
October	**+ **9.01	**+ **0.15	**+ **0.67	**+ **0.10	**+ **0.77	**+ **0.04	**+ **0.07	0.03
November	**+ **3.79	**+ **0.09	**+ **0.48	**+ **0.09	**+ **0.52	**+ **0.06	**+ **0.07	**+ **0.01
December	**+ **3.32	**+ **0.06	**+ **0.32	**+ **0.07	**+ **0.36	0.00	**+ **0.15	**+ **0.05

Total erosivity in MJ mm ha^-1^ h^-1^ month^-1^; Total kinetic energy in MJ ha^-1^ month^-1^; Total erosive rainfall in mm month^-1^;  Maximum 60-min intensity in mm h^-1^ month^-1^; Total rainfall in mm month^-1^; Monthly erosivity density in MJ mm ha^-1^ h^-1^ mm^-1^.

For all other attributes, increases were more common and were found to be significant in two to four months, suggesting that all attributes other than the number of events followed a similar trend (see Supplementary Table 7). A notable seasonal variation in the trends was observed—out of the 33 significant cases, 23 occurred between October and January, a period corresponding to the post-monsoon and winter seasons when overall erosivity is typically low. This was particularly evident in monthly erosivity, where increases during these months have limited relevance to the total annual erosivity.

However, after applying False Discovery Rate (FDR) corrections to control for multiple testing, 23 out of the 33 initially significant trends were found to be statistically insignificant. Only 10 cases remained significant (see adjusted p-values in Supplementary Table 7), and most of these were found in the post-monsoon and winter months, which are generally non-erosive.

This seasonal clustering of significant changes, mainly in low-erosivity months, may reflect subtle shifts in rainfall intensity or timing, potentially driven by changing climate dynamics. In contrast, the peak monsoon season (June to September)—which contributes the most to annual erosivity—remained relatively stable with fewer significant changes.

## Discussion

### Comparison with the global dataset

**Fig. 5:**
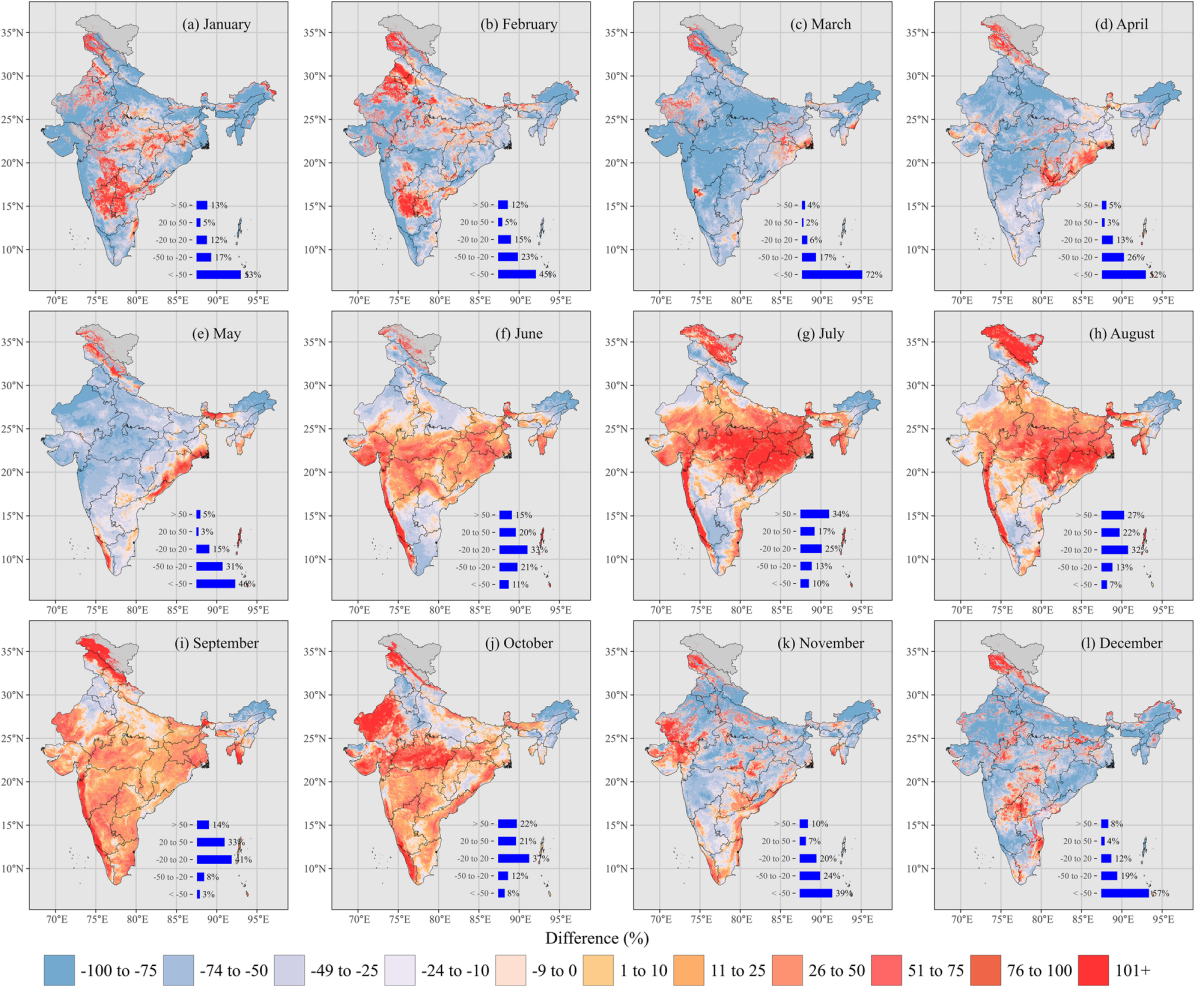
Percentage error (%) of long-term, highly resolved monthly erosivity estimates from this study compared to the Global Rainfall Erosivity Dataset version 1.2 (GloREDa v1.2)^90^. Negative values indicate that GloREDa v1.2 estimates are higher than those in this study, while positive values indicate lower estimates in GloREDa v1.2 relative to this study. The maps were generated using R version 4.4.3 with the packages *ggplot2*, *sf*, and *raster.*

We compared the estimated monthly erosivity at a ~ 1 km^2^ resolution with the Global Rainfall Erosivity Dataset version 1.2 (GloREDa v1.2)^90^ for each month (Fig. 5). Large portions of India exhibit negative differences during the pre-monsoon months (March to May) (Fig. 5c–e), with this study’s erosivity estimates being lower than GloREDa by more than -50% in Northeast India and the Eastern Ghats. However, certain regions, such as the Central Highlands, show positive differences, where estimated erosivity exceeds GloREDa estimates.

In May, negative differences persist in the Thar Desert region and central Highlands, ranging from − 75% to − 25%, while positive differences (up to + 75%) are observed along the Eastern Ghats. Significant positive differences are seen during the peak monsoon months (June to September) (Fig. 5f-i), with erosivity in the study area exceeding GloREDa estimates by + 20% to more than + 50% in Central Highlands and the Western Ghats. By September and October (Fig. 5i and j), erosivity differences begin to decrease, showing a mix of negative and positive differences. In the winter season (Fig. 5a, b, and l), stronger negative differences appear in the plains, but positive differences are noted in the western Himalayas and the Deccan Plateau regions.

Overall, our results indicate that the rainfall erosivity estimates from this study tend to be lower during the dry months and higher during the monsoon months (Fig. 5c–k), when compared to GloREDa v1.2. This variation suggests that global datasets such as GloREDa may underestimate the local erosive power of intense monsoon rainfall, largely due to limited observational data (e.g., reported in earlier studies^32,119–121^). Additionally, GloREDa v1.2, an extension of the first version of GloREDa, employs a different kinetic energy equation than that used in this study. Moreover, the rainfall dataset utilized in GloREDa is shorter in duration, potentially failing to capture long-term climatic variability^78,122^. This issue is particularly relevant in India, where significant changes in climate and rainfall patterns have occurred in recent decades^123^. Therefore, local and regional-scale studies are essential for accurately identifying and understanding erosivity patterns across both spatial and temporal scales.

### Comparison with past rainfall erosivity equations

The history of erosivity studies in India involves the use of various empirical equations. Most of them used the metric version of these equations until newer converted formulas were developed (Table 4). Alongside the erosivity equations of Babu et al.^34,35^, another popular approach based on the modified Fournier equation^33,42^ has also been used for erosivity estimation in many studies. Initially formulated in metric units, these equations were converted to the SI unit system by Majhi et al. ^26^ and Chen et al.^24^.We estimated erosivity using these equations and compared the results with our observed in-situ erosivity values presented in the Supplementary Fig. 17. The results indicate that these equations tend to underestimate rainfall erosivity in most cases. Specifically, the equation of Babu et al^35^ even after converting to SI unit by Majhi et al.^26^ shows a mean percentage error of almost − 35% across India, with a median of almost − 42%. Notably, the unit-converted equation of Arnoldus’s ^33^ has a mean percentage error of − 9%, with a median of -17%, though it results in both positive and negative errors ranging from − 75% to + 75%.

**Table 4:** Comparison of earlier used erosivity equations in India with this study.

Eq. No	Original equation	Unit converted equation (*R* in MJ mm ha^−1^ h^−1^ per year or season) and rainfall range	Correlations	Study
Annual erosivity				
17	$R = 1.735 \times 10^{{(1.5\log_{10} \left( {\mathop \sum \limits_{i = 1}^{12} \frac{{P_{i}^{2} }}{P}} \right) - 0.8188)}}$ $P_{i}$: rainfall of month *i* (mm month^−1 ^); *R *in metric units	$R = 17.02 \times 10^{{(1.5\log_{10} \left( {\mathop \sum \limits_{i = 1}^{12} \frac{{P_{i}^{2} }}{P}} \right) - 0.8188)}}$	n.a	#1
18	$R = 79 + 0.363 P$ *R* in t-m cm ha^−1 ^ h^−1 ^ year^−1 ^	$R = 3.70 P + 806$ Rainfall range: 350 to 3500 mm year^−1 ^	0.69	#2
20	$R = 81.5 + 0.380 P$ *R* in t-m cm ha^−1 ^ h^−1 ^ year^−1 ^	$R = 3.88 P + 832$ Rainfall range: 340 to 3500 mm year^−1 ^	0.81	#3
22	$R = 14.41 P - 6514$ *R* in MJ mm ha^−1 ^ h^−1 ^ year^−1 ^; Rainfall range: 226 to 8946 mm year^-1^		0.92	#4
Summer monsoon erosivity				
19	$R_{s} = 50 + 0.389 P_{s}$ *R* in t-m cm ha^−1 ^ h^−1 ^ season^−1 ^	$R_{s} = 3.97 P_{s} + 510$ Rainfall range: 300 to 3000 mm season^−1 ^	0.77	#2
21	$R_{s} = 71.9 + 0.361 P_{s}$ *R* in t-m cm ha^−1 ^ h^−1 ^ season^−1 ^	$R_{s} = 3.68 P_{s} + 734$ Rainfall range: 293 to 3190 mm season^−1 ^	0.82	#3
23	$R_{s} = 14.61 P_{s} - 4693$ *R*_s_ in MJ mm ha^−1 ^ h^−1 ^ year^−1^; Rainfall range: 32 to 7448 mm season^-1^		0.92	#4

P, Long-term average annual rainfall; $P_{s}$, Long-term average summer-monsoon rainfall; R, Long-term average annual erosivity; $R_{s}$, Long-term average erosivity for summer monsoon season.

Studies:

#1 Original version: Arnoldus’s^33^, units converted by Chen et al.^24^,

#2 Original version: Babu et al^34^ as cited in Singh^53^ united converted using the procedure of Majhi et al.^26^,

#3 Original version: Babu et al^35^, units converted by Majhi et al.^26^,

#4 This study

Additionally, when we applied a simplified rainfall erosivity equation based solely on rainfall data from 261 stations (Eqs. 22 and 23), we observed a low percentage error at the annual level; however, a high seasonal error was evident. Furthermore, the rainfall erosivity map prepared in this study showed a median percentage error of − 20% even after interpolation at the annual scale, with a mean error of only − 18%. Notably, the error is lower for the summer monsoon months, with a median percentage error of only − 18%. Therefore, the combination of the machine learning model and GWR-based interpolation demonstrated strong performance compared to previous studies. In contrast, the empirical rainfall erosivity equations developed in earlier works are now outdated and tend to introduce significant bias across India. Several regional studies—for instance, Pandey et al.^124^, which estimated erosion in Indian forests, and Pal et al.^125^, which applied these equations for large-scale analyses—are likely to have incurred substantial errors in rainfall erosivity estimation and, consequently, in erosion estimation.

Hence, the use of such empirical equations should be approached with caution. Moreover, long-term erosivity estimates derived from these equations may vary depending on the temporal coverage of the data used to develop the equations and the period for which they are applied, which can potentially lead to significant differences in the results. We strongly recommend utilizing hourly rainfall data from the India Meteorological Department (IMD) for more accurate and reliable erosion assessments. Furthermore, despite the limited availability of hourly station data, the integration of machine learning and artificial intelligence with geo-climate variables or satellite datasets now makes it possible to estimate accurate rainfall erosivity in data-sparse regions or areas with complex topography. This approach will eventually eliminate the reliance on regionally developed empirical equations and facilitate more accurate estimation of rainfall erosivity for specific time periods and locations.

### Error analysis

There are notable sources of error that may affect this study’s results. One is the use of WorldClim maximum and minimum temperature data for stations lacking observed records—excluding the 510 stations with observations. To assess the reliability of this method, we compared observed temperatures with WorldClim data at the same locations. The monthly median percentage error remains low—around ± 1% for maximum temperature (Supplementary Fig. 18), and slightly higher for minimum temperature, with + 3.77% in January and + 3.11% in February (Supplementary Fig. 19). All errors remain within ± 4%, which may partly explain the higher prediction errors noted in these months. Furthermore, similar error ranges in the WorldClim dataset have been reported in previous studies for Europe ^126^, global scale^127^, and Nepal^128^. Given the unavailability of observed temperature records for all stations, we believe that using the WorldClim dataset is a reasonable and justifiable alternative despite these minor errors.

Another source of uncertainty is the use of correction factors to convert hourly rainfall erosivity to a 1-min scale, which inherently omits peak 30-min intensity. We compared our adopted factor with those from other studies (Supplementary Table 8), showing errors ranging from − 40.67% to + 73.61%. However, our results align well, showing only − 3.89% error with Panagos et al^129^. (Europe), + 6.89% with Yue et al^130^. (China), and − 2.44% with Fischer et al^80^. (Germany). Given the lack of breakpoint pluviograph or 1-min rainfall datasets in many regions, our adopted correction factor offers a reliable and practical approach for utilizing hourly data in rainfall erosivity estimation.

Furthermore, we analyzed the variability in prediction errors (residuals) of erosivity in relation to key controlling factors—rainfall amount, elevation, land use classes, and soil types—using ANOVA. The detailed results are provided in Supplementary Table 9. The analysis revealed a clear seasonal pattern in the residuals. Specifically, rainfall significantly affected residuals during winter or dry periods (*p* < 0.05), and similar higher rainfall erosivity variability under low rainfall conditions was observed in Europe^84^. Elevation and land use classes showed significant impacts during the winter and pre-monsoon months, while soil types influenced residuals primarily during the summer monsoon season. A similar topographic influence on rainfall erosivity has also been reported for the Tibetan Plateau^131^.

### Limitations and future scope

The study has several limitations due to the limited availability of high-resolution datasets in a tropical country like India. Variations in the start and end years of hourly and monthly rainfall across stations introduce inconsistencies in temporal coverage, which may affect the accuracy of erosivity estimates. Despite these challenges, we have attempted to incorporate long-term rainfall datasets to derive long-term average erosivity values. However, climate variability—such as shifts in rainfall patterns, frequency, and intensity—can significantly influence these results^132^. Additionally, recent studies have highlighted an increase in extreme rainfall events and changing monsoonal behavior in South Asia, driven by both natural variability and anthropogenic influences^133^. These changes may render historical erosivity equations outdated, especially those developed using short-term or localized datasets. Therefore, it is essential to rely on updated observed data and high-resolution gridded products to capture contemporary erosivity dynamics more accurately.

The reliance on WorldClim data to fill gaps in temperature records may introduce uncertainties due to resolution differences. While the error analysis shows that the median error is low, the error range is considerably larger, particularly in January and February. However, future studies could benefit from incorporating more accurate datasets, such as CHELSA^127^, or satellite-based and reanalysis products, or even combinations of these, which could help reduce interpolation errors and improve erosivity assessments.

Additionally, the conversion factors used to estimate 1-min erosivity from 60-min data are approximations that may not fully capture storm variability^75,80,119^. The lack of high temporal resolution datasets hinders the accurate estimation of maximum 30-min intensity, which can lead to under- or overestimation of rainfall erosivity values, particularly in regions with intense rainfall, such as the Western Ghats and Northeast India. We encourage researchers to derive region-specific correction factors using high-temporal resolution datasets to improve accuracy (e.g., Yue et al.^130^). Furthermore, future studies are encouraged to incorporate high-temporal resolution satellite and climate reanalysis datasets, along with limited gauge observations (e.g., Yonaba et al.^30^), to eliminate the need for such conversion factors in erosivity estimation.

Furthermore, the performance of the XGBoost machine learning model also depends on data availability, which could impact its generalizability^134^. Although we employed a two-way validation approach to improve the robustness of the model, XGBoost and similar machine learning models are still prone to overfitting and are highly sensitive to the structure of the input data. Future studies are recommended to explore advanced modeling techniques such as Deep Learning models^135^ (e.g., Artificial Neural Networks, Long Short-Term Memory, and Convolutional Neural Networks), which are often more resilient to overfitting and capable of capturing complex spatio–temporal patterns.

While GWR captures spatial non-stationarity effectively, it is sensitive to data density, multicollinearity, and bandwidth selection^134^. The model may produce unstable estimates in sparsely gauged regions, and its results can be difficult to interpret due to spatially varying coefficients (e.g., high interpolation error for the winter months). Meusburger et al.^89^ also observed that in Switzerland, rainfall erosivity values were lowest during winter months, leading to increased uncertainty in spatial predictions. Future studies are encouraged to use recently available high temporal resolution radar remote sensing datasets (e.g., Dai et al.^136^, Auerswald et al.^75^) which can be applied on a broader scale and reduce the interpolation error.

Notably, the gap-filled SRTM dataset used in this study, obtained from the CGIAR-CSI, has certain limitations in accurately capturing ground elevation. As demonstrated in past studies^137,138^, although the CGIAR-CSI dataset offers improved vertical accuracy compared to standard SRTM data, the enhancements in slope and aspect measurements are significant only for slopes greater than 10°. For slopes less than 10°, however, the improvement is not as pronounced. Future studies are encouraged to use regionally available higher-resolution topographic information for event improvement in the DEM, such as ASTER-GDEM or CartoDEM, to achieve more accurate erosivity estimation.

The high-resolution mapping of rainfall erosivity in a traditional way requires more data, specifically at least one observation for each raster grid^139^. However, such detailed observations are likely unavailable for India. Even many countries in the Global South rarely have such high-temporal resolution and spatially dense datasets for rainfall erosivity and erosion estimation. We believe that limited observations of rainfall datasets, along with the help of machine learning and artificial intelligence, could significantly improve large-scale erosivity estimation. We strongly recommend that future studies address these limitations to improve accuracy. These limitations should be considered when interpreting the study’s results, and future research could focus on overcoming these challenges.

## Conclusion

The analysis of monthly erosivity across India reveals a clear spatio–temporal pattern, with much of the country experiencing significantly high erosivity during the summer monsoon months (June to September). Notably, July sees the peak erosivity, primarily driven by total rainfall, with most of the rainfall occurring in large events classified as erosive. Interestingly, the number of erosive events exceeded 15 for many stations between June and August, indicating that an erosive event occurred at least every second day. This pattern was particularly evident in July and August, with one-quarter of all stations experiencing such frequent erosive events.

In contrast, the remaining months (October to May) exhibit consistently lower erosivity values, except along the southern coast and Himalayan ranges, where erosive rainfall also occurs during the pre- and post-monsoon periods. The maximum 60-min rainfall intensity during the summer monsoon can reach up to 50 mm h^−1 ^, while in the rest of the year, it typically remains below 20 mm h^−1 ^.

The spatial patterns in rainfall erosivity during the summer monsoon months are clearly dominated by rainfall and elevation patterns. The SHAP analysis reveals that rainfall, surface runoff, and water vapor pressure are the primary drivers of monthly rainfall erosivity across India, with their influence varying seasonally. In contrast, factors like soil moisture, wind speed, slope, soil type, and land use play a minimal role in erosivity estimation at the national scale. Distance from the coast showed moderate importance, particularly during the pre-monsoon and winter seasons, due to its impact on regional moisture availability and atmospheric dynamics.

Long-term trends from 1969 to 2021 reveal a slight increasing trend in monthly erosivity for most months, except for a decreasing trend in August. However, only the trend for January (+ 0.86 MJ mm ha^−1 ^ h^−1 ^ month^−1 ^per year) was found to be statistically significant. Notably, among the 96 trend tests (12 months × 8 attributes), only 10 exhibited statistically significant trends. A statistically significant increase in the 60-min rainfall intensity was observed during the post-monsoon and winter months, with an annual increase rate of approximately + 0.1 mm h^−1 ^.

Several sources of uncertainty in the current study warrant consideration—such as the reliance on WorldClim temperature data for stations lacking observations and the use of correction factors to estimate 1-min rainfall erosivity from hourly data. Although these approaches showed acceptable validation errors against observed and literature-based datasets, they may still affect estimation accuracy in months with high variability. Future research should prioritize the integration of high-temporal resolution satellite data, climate reanalysis products, and improved ground-based datasets such as CHELSA. This would help reduce the uncertainty and enhance the precision of erosivity estimates. These improvements will ultimately strengthen the basis for developing effective, region-specific soil conservation and erosion management strategies—particularly during the monsoon season when erosivity peaks.

## Supplementary Information

Supplementary Information.

## Data Availability

The dataset set can be made available from the corresponding author on a reasonable request.
