# Supplementary material for: Identifying monthly rainfall erosivity patterns using hourly rainfall data across India
Source: Sci Rep. 2025 Jul 31;15:27940. doi: 10.1038/s41598-025-11992-x (PMC12314091; doi:10.1038/s41598-025-11992-x)
Supplement: Supplementary file 1 — Supplementary Information. [file 41598_2025_11992_MOESM1_ESM.docx]

**Supplementary material for:** **Identifying monthly rainfall erosivity patterns using hourly rainfall data across India**

Subhankar Das^1,*^, Manoj Kumar Jain^1^, Karl Auerswald^2^, Carlos Rogerio de Mello^3^, Peter Molnar^4^

^1^ Department of Hydrology, Indian Institute of Technology Roorkee, Roorkee, India.

^2^ School of Life Sciences, Technical University of Munich, Freising, Germany.

^3^ Water Resources Department, Federal University of Lavras, Lavras, Brazil.

^4^ Department of Civil, Environmental and Geomatic Engineering, ETH Zürich, Zürich, Switzerland

*Corresponding author Email: [sdas@hy.iitr.ac.in](mailto:sdas@hy.iitr.ac.in)


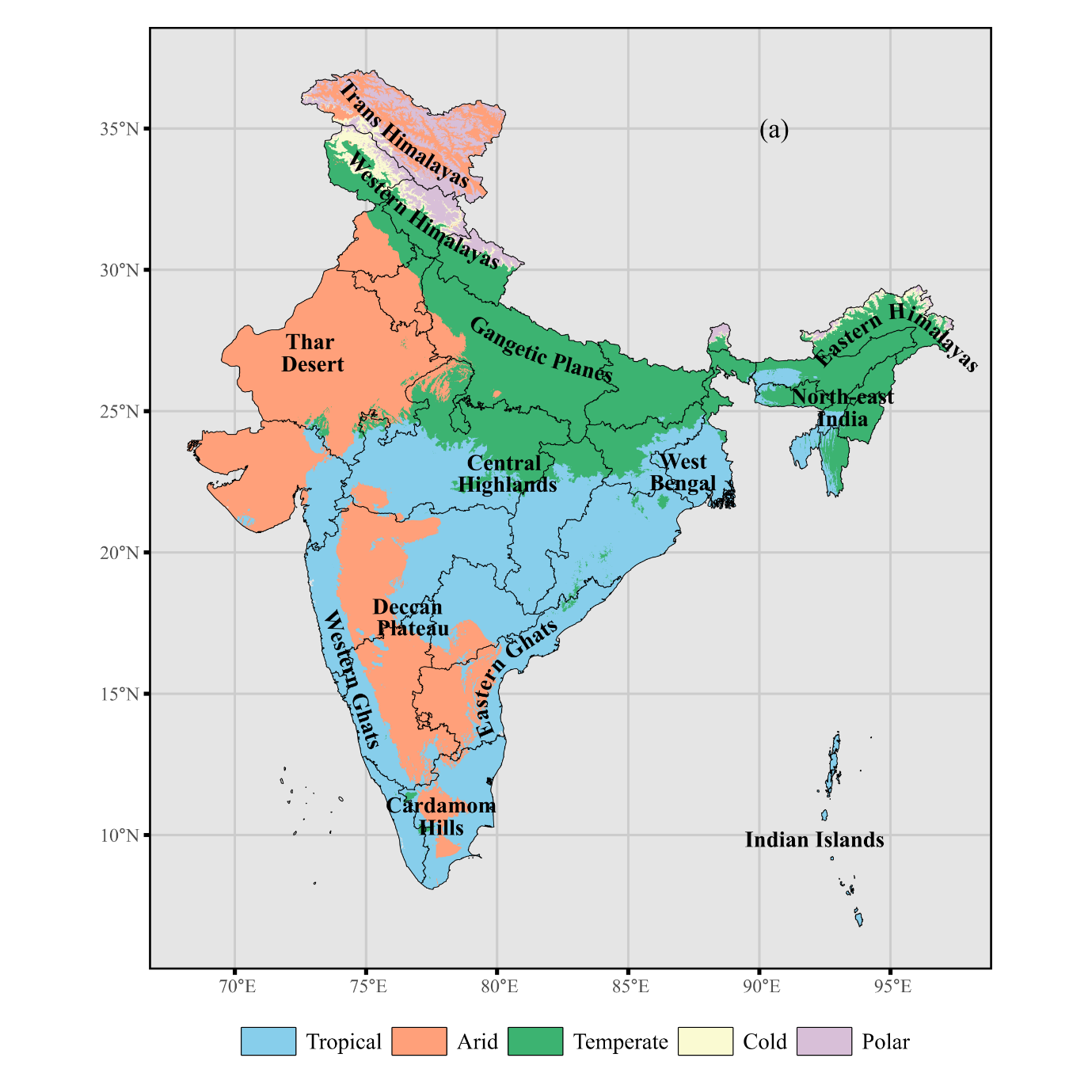


**Supplementary Figure 1.** Climate map of India from the Koppen-Geiger climate classification map at 1-km resolution ^1^**.** The map was generated using R version 4.4.3 with the ggplot2, sf, and raster packages.

**Supplementary Figure 2.** Correlation matrix illustrates the relationship between rainfall erosivity, and geo-climatic variables used in the XGBoost model for January. The plots were generated using R version 4.4.3 with the package ggplot2.





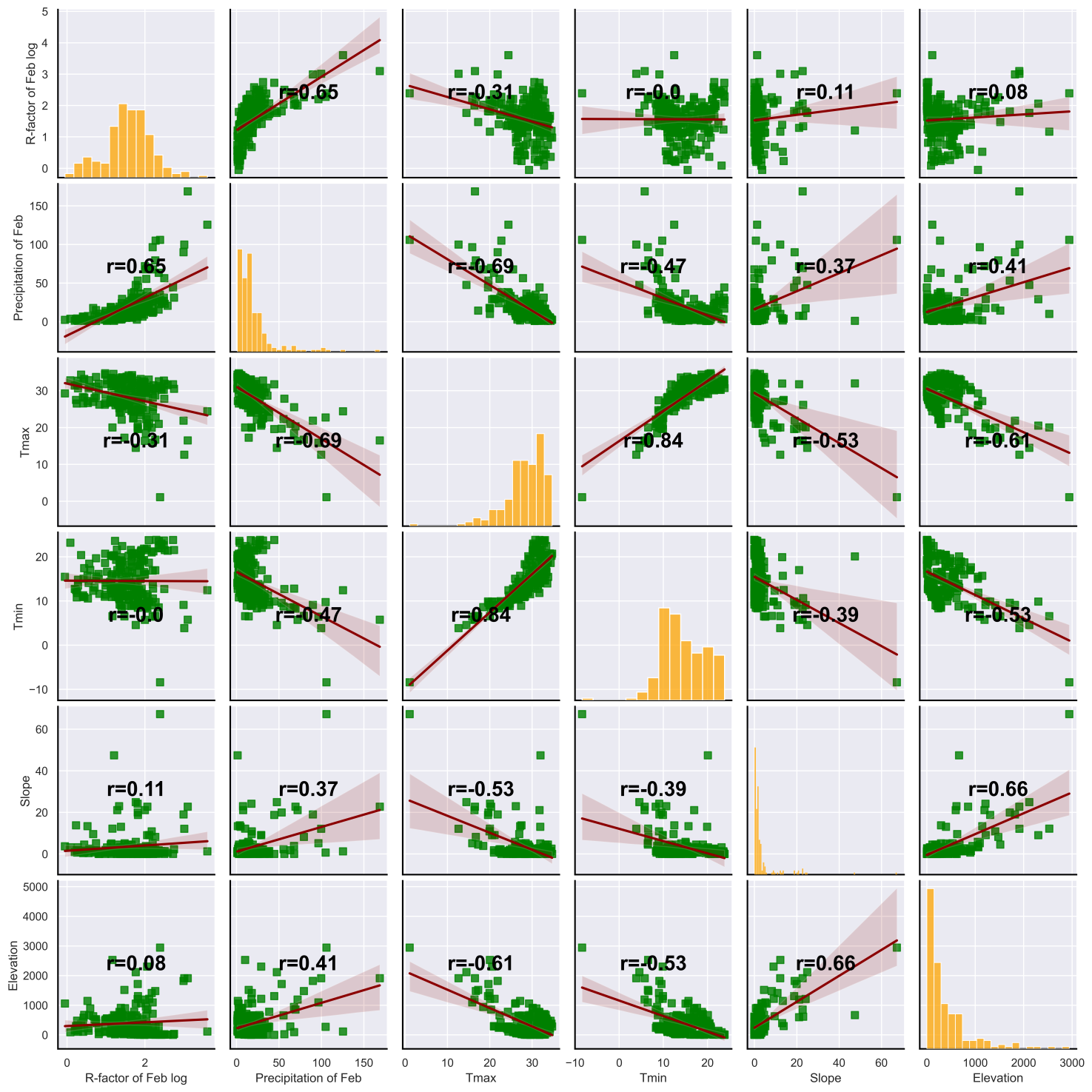


**Supplementary Figure 3.** Correlation matrix illustrates the relationships between rainfall erosivity, and geo-climatic variables used in the XGBoost model for February. The plots were generated using R version 4.4.3 with the package ggplot2.


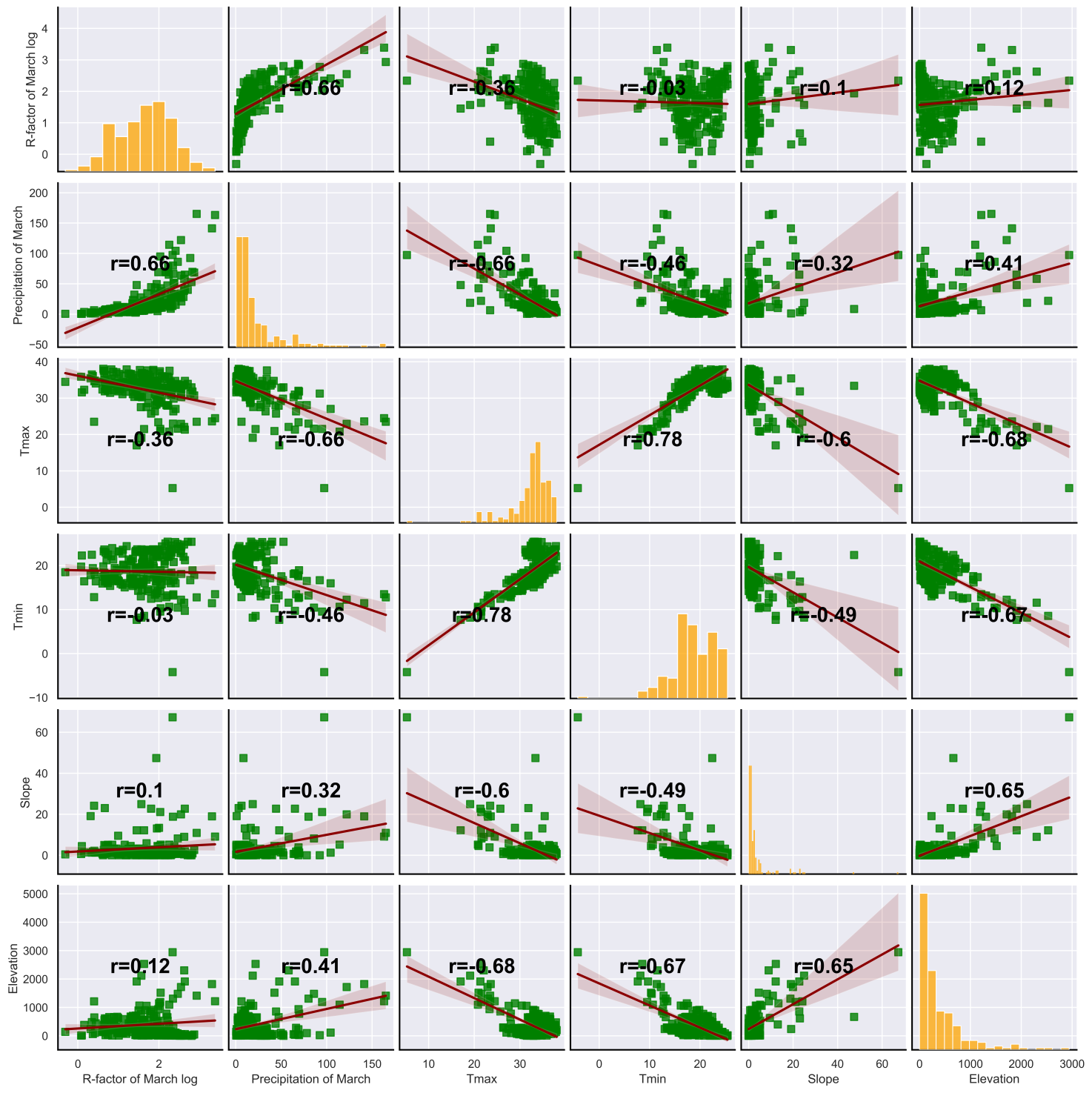


**Supplementary Figure 4.** Correlation matrix illustrates the relationship between rainfall erosivity, and geo-climatic variables used in the XGBoost model for March. The plots were generated using R version 4.4.3 with the package ggplot2.


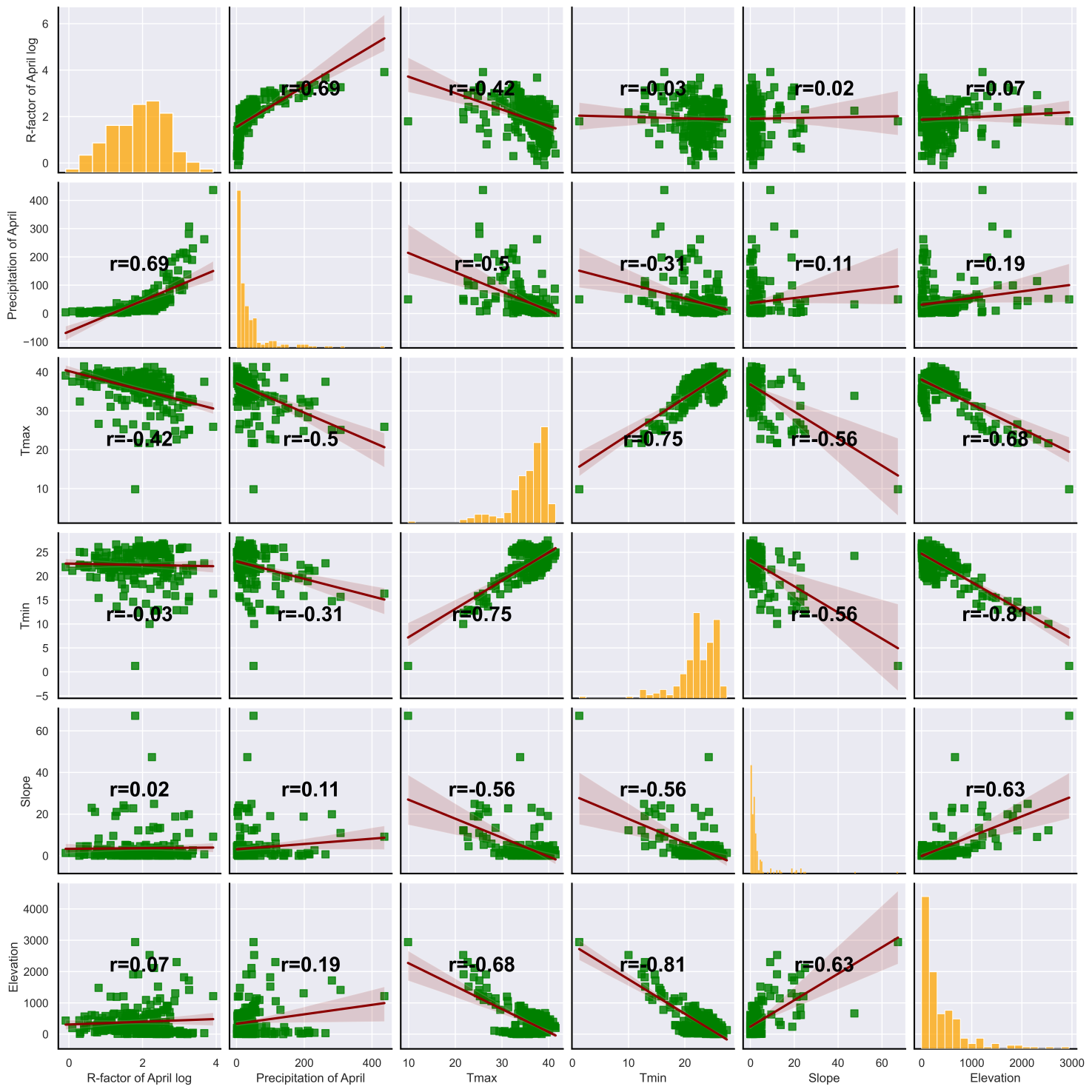


**Supplementary Figure 5.** Correlation matrix illustrates the relationship between rainfall erosivity, and geo-climatic variables used in the XGBoost model for April. The plots were generated using R version 4.4.3 with the package ggplot2.


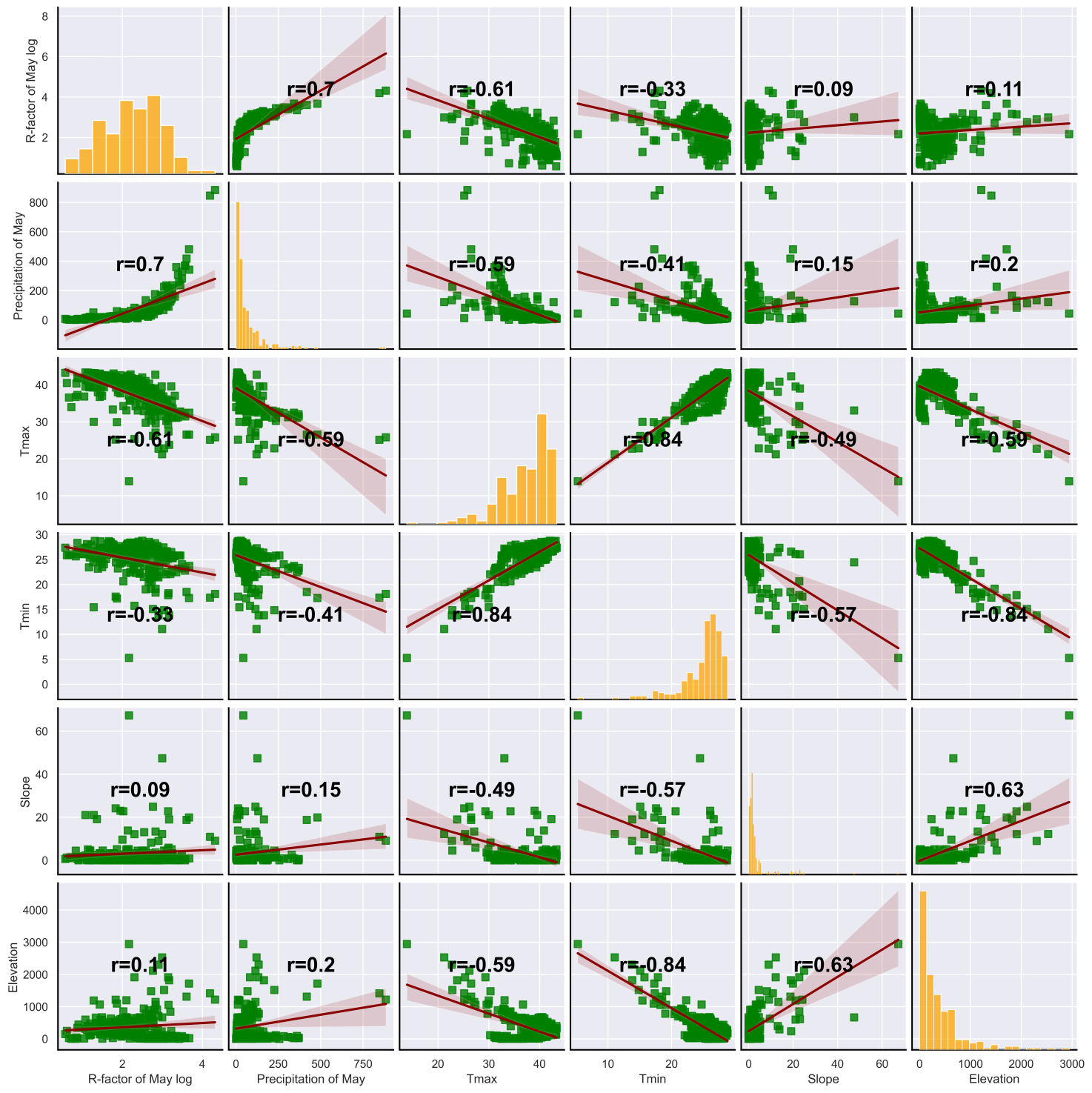


**Supplementary Figure 6.** Correlation matrix illustrates the relationships between rainfall erosivity, and geo-climatic variables used in the XGBoost model for May. The plots were generated using R version 4.4.3 with the package ggplot2.


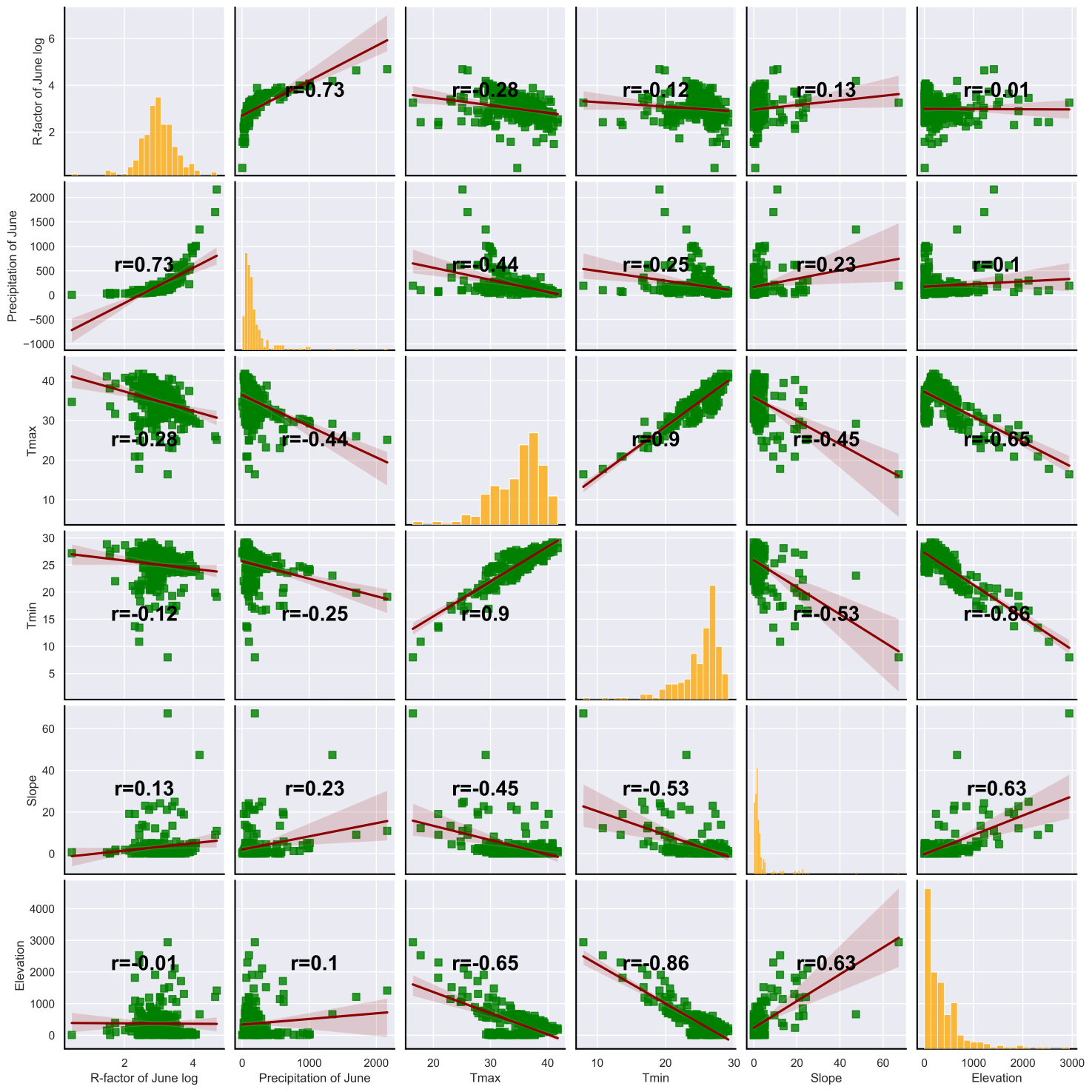


**Supplementary Figure 7.** Correlation matrix illustrates the relationship between rainfall erosivity, and geo-climatic variables used in the XGBoost model for June. The plots were generated using R version 4.4.3 with the package ggplot2.


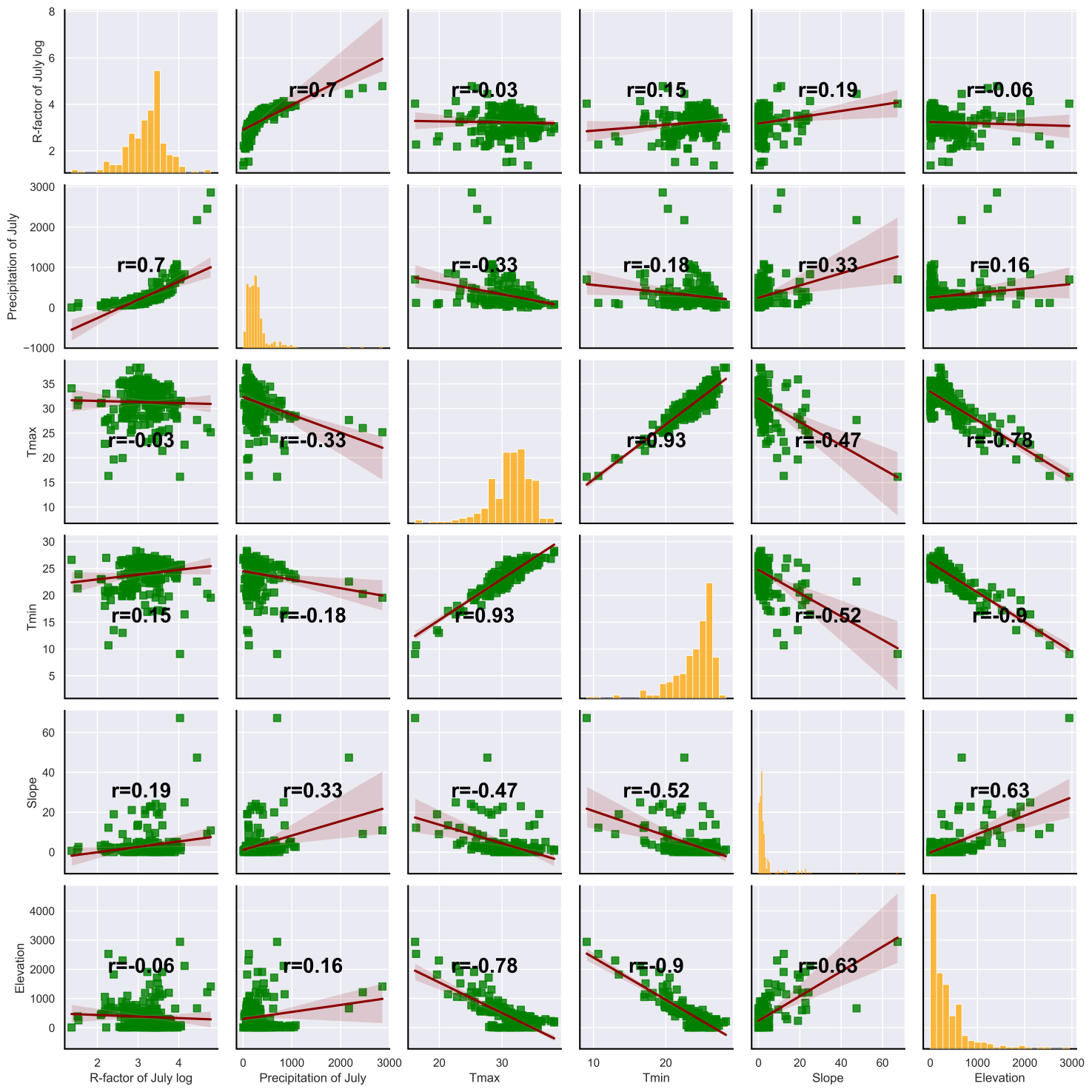


**Supplementary Figure 8.** Correlation matrix illustrates the relationship between rainfall erosivity, and geo-climatic variables used in the XGBoost model for July. The plots were generated using R version 4.4.3 with the package ggplot2.


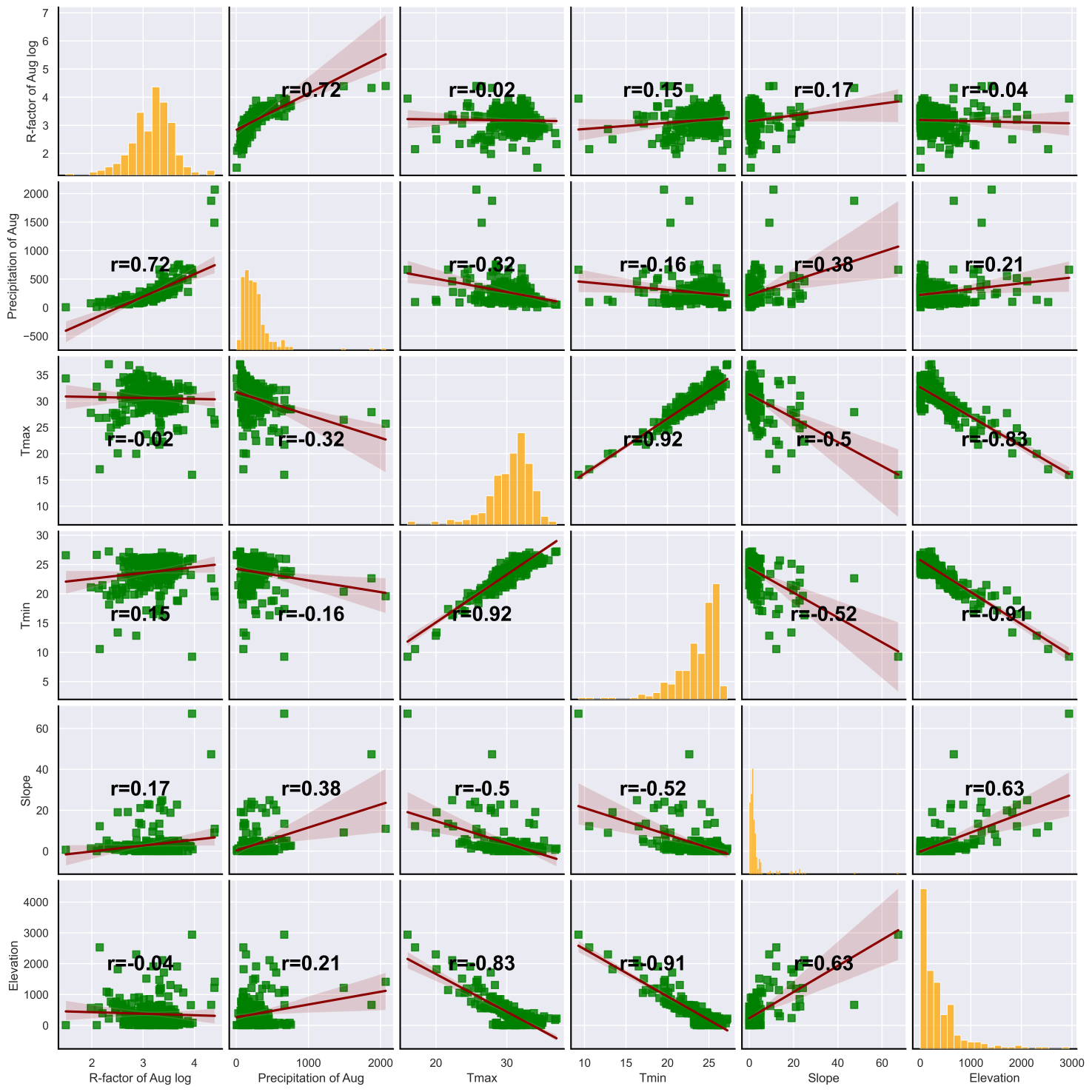


**Supplementary Figure 9.** Correlation matrix illustrates the relationships between rainfall erosivity, and geo-climatic variables used in the XGBoost model for August. The plots were generated using R version 4.4.3 with the package ggplot2.


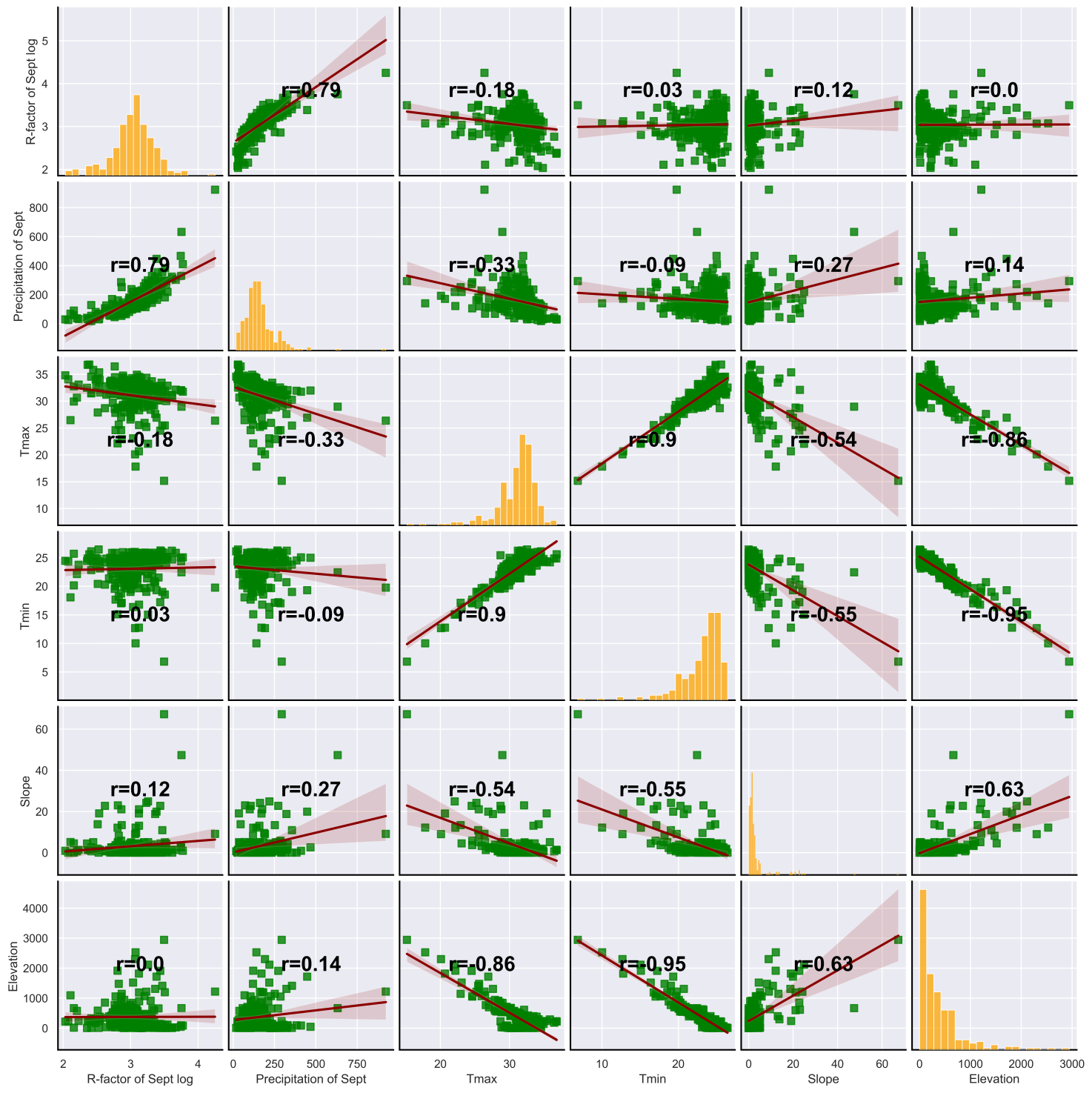


**Supplementary Figure 10.** Correlation matrix illustrates the relationship between rainfall erosivity, and geo-climatic variables used in the XGBoost model for September. The plots were generated using R version 4.4.3 with the package ggplot2.


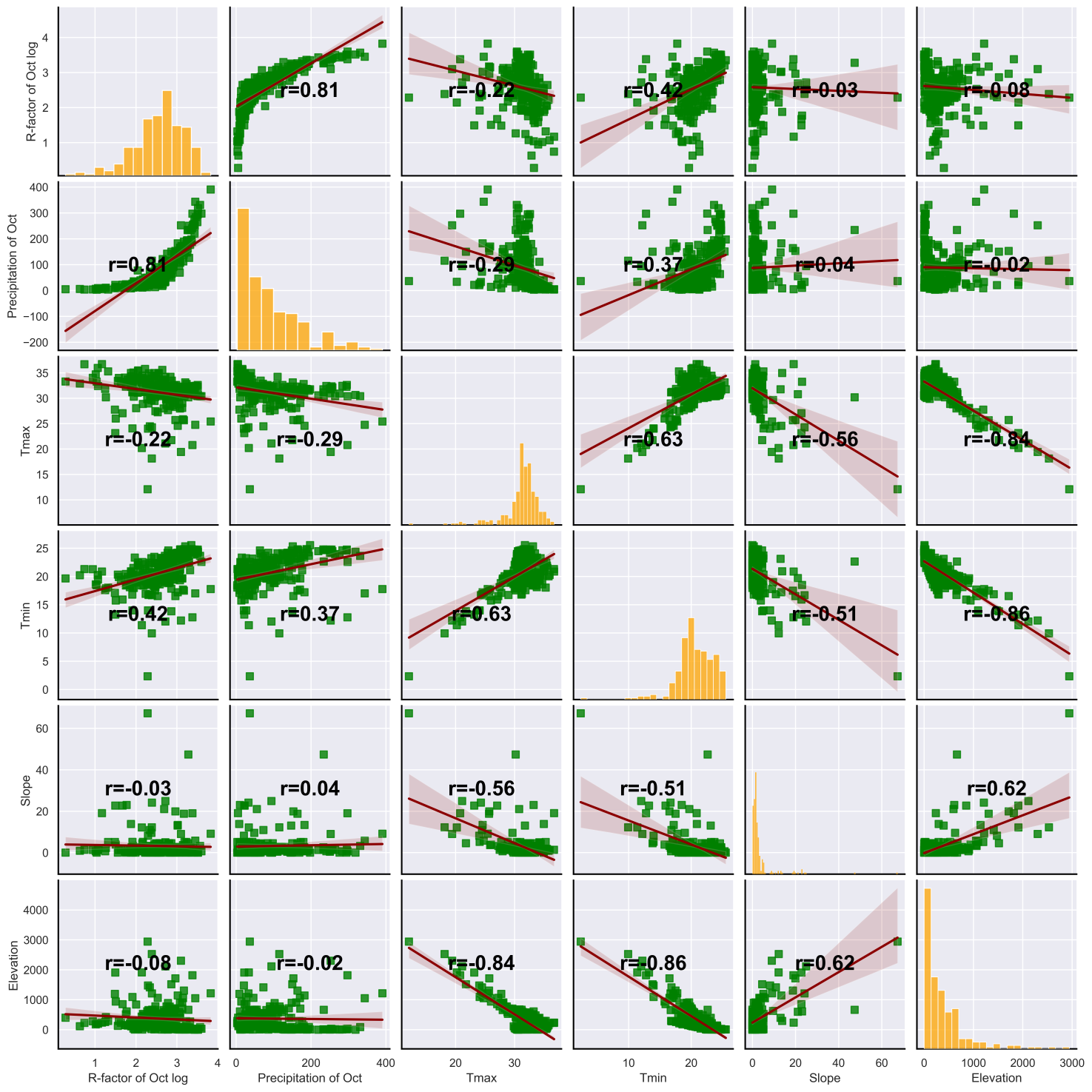


**Supplementary Figure 11.** Correlation matrix illustrates the relationship between rainfall erosivity, and geo-climatic variables used in the XGBoost model for October. The plots were generated using R version 4.4.3 with the package ggplot2.


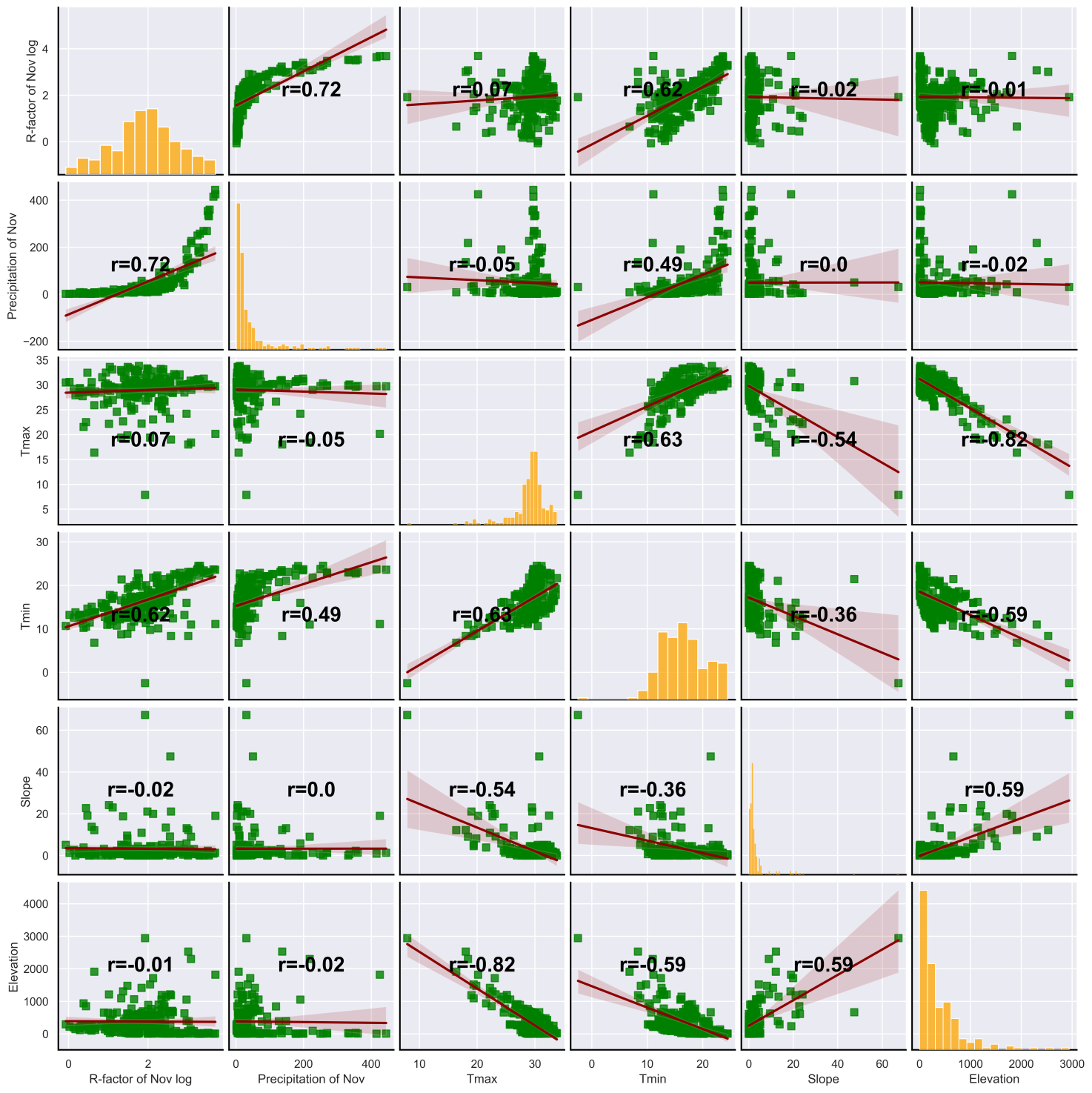


**Supplementary Figure 12.** Correlation matrix illustrates the relationships between rainfall erosivity, and geo-climatic variables used in the XGBoost model for November. The plots were generated using R version 4.4.3 with the package ggplot2.


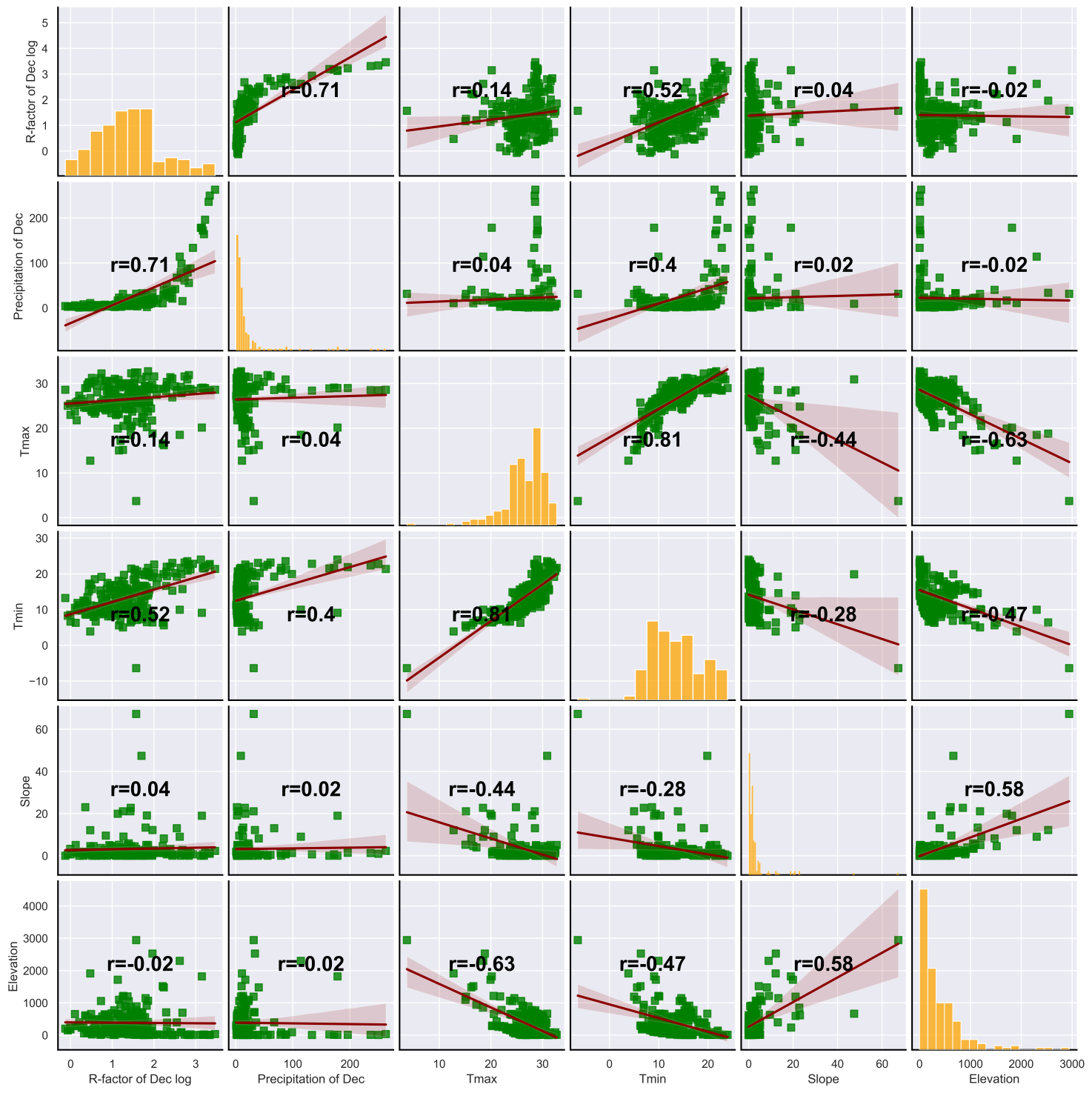


**Supplementary Figure 13.** Correlation matrix illustrates the relationship between rainfall erosivity, and geo-climatic variables used in the XGBoost model for December. The plots were generated using R version 4.4.3 with the package ggplot2.


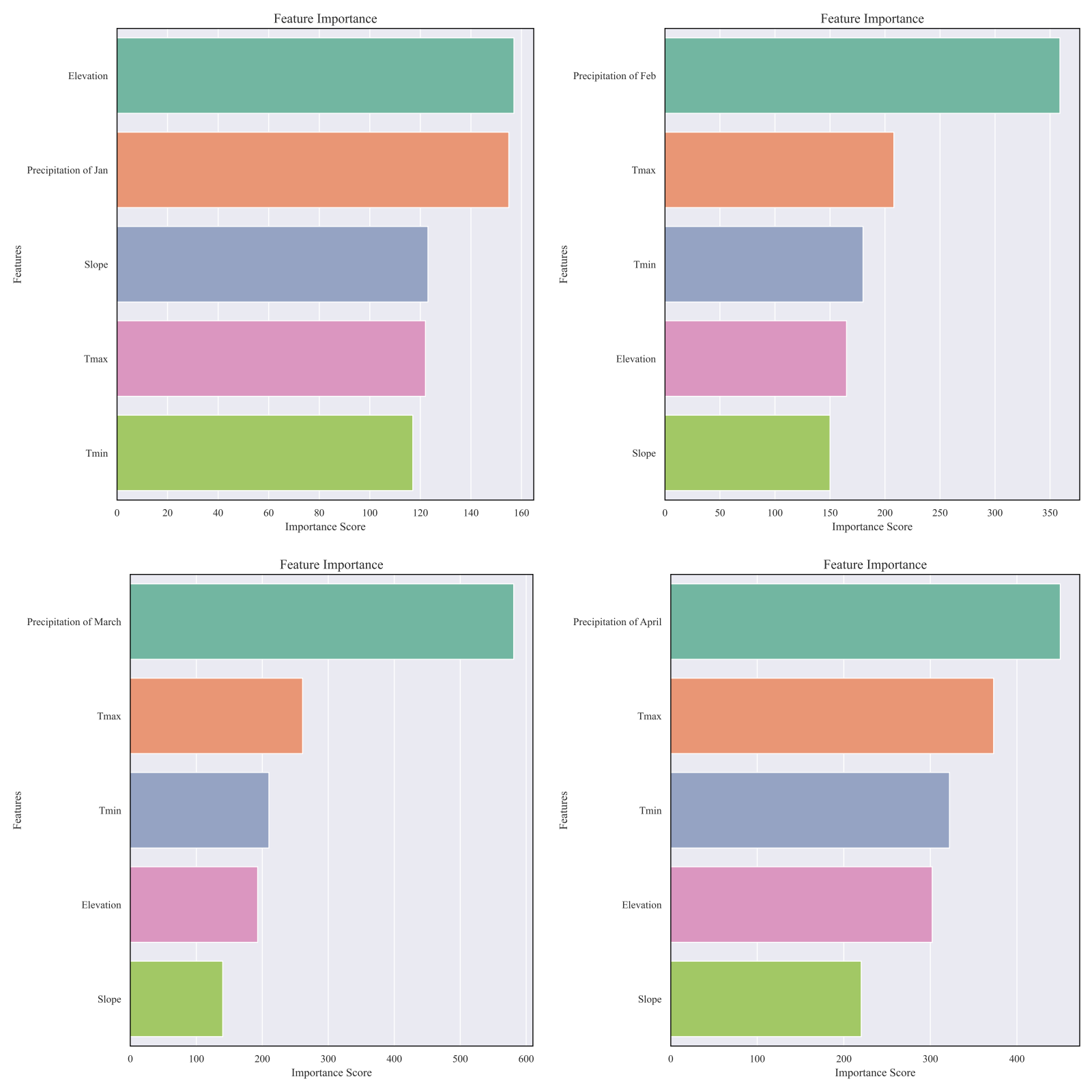


**Supplementary Figure 14.** Feature importance plot for the monthly XGBoost model from January to April. The plots were generated using R version 4.4.3 with the package ggplot2.


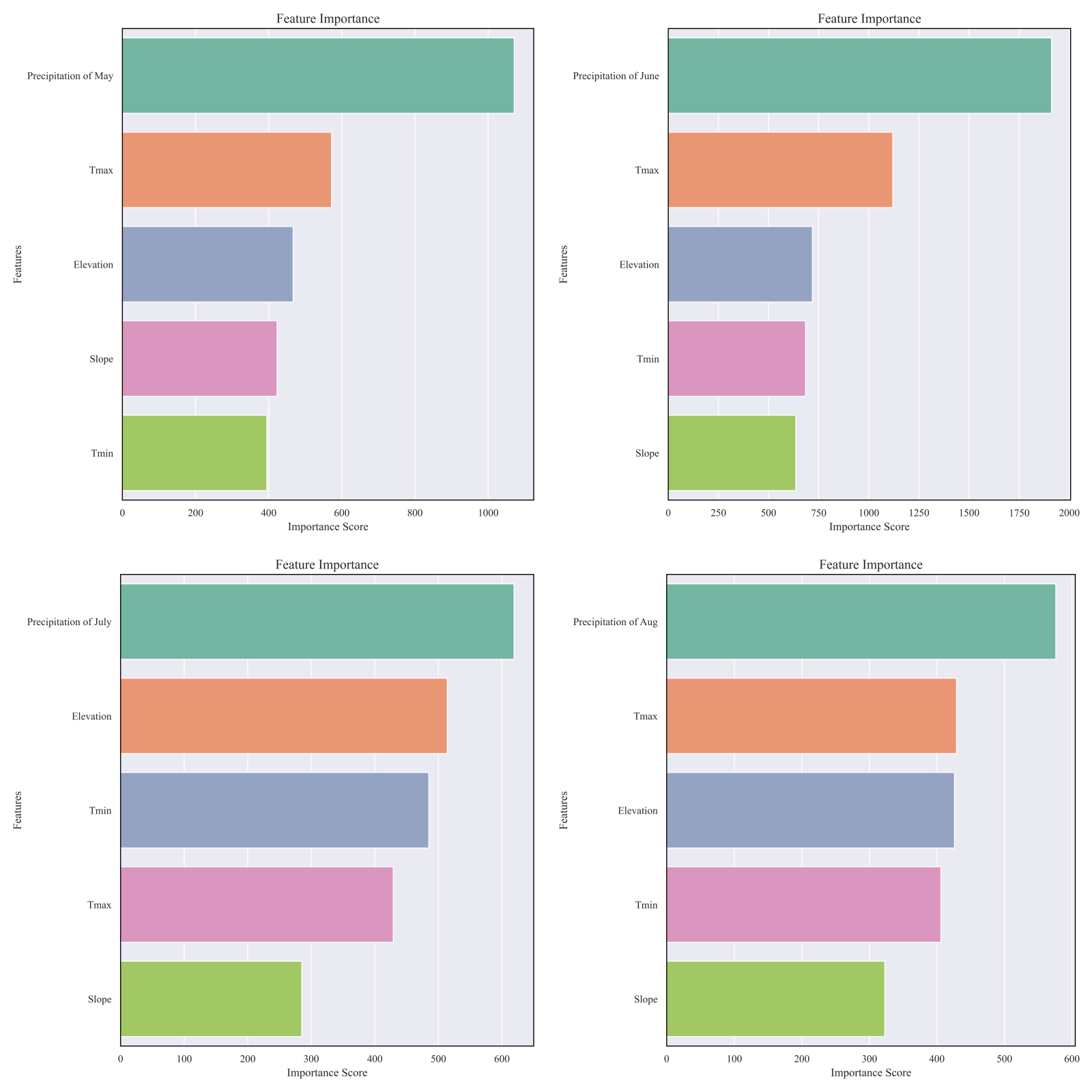


**Supplementary Figure 15.** Feature importance plot for the monthly XGBoost model from May to August. The plots were generated using R version 4.4.3 with the package ggplot2.


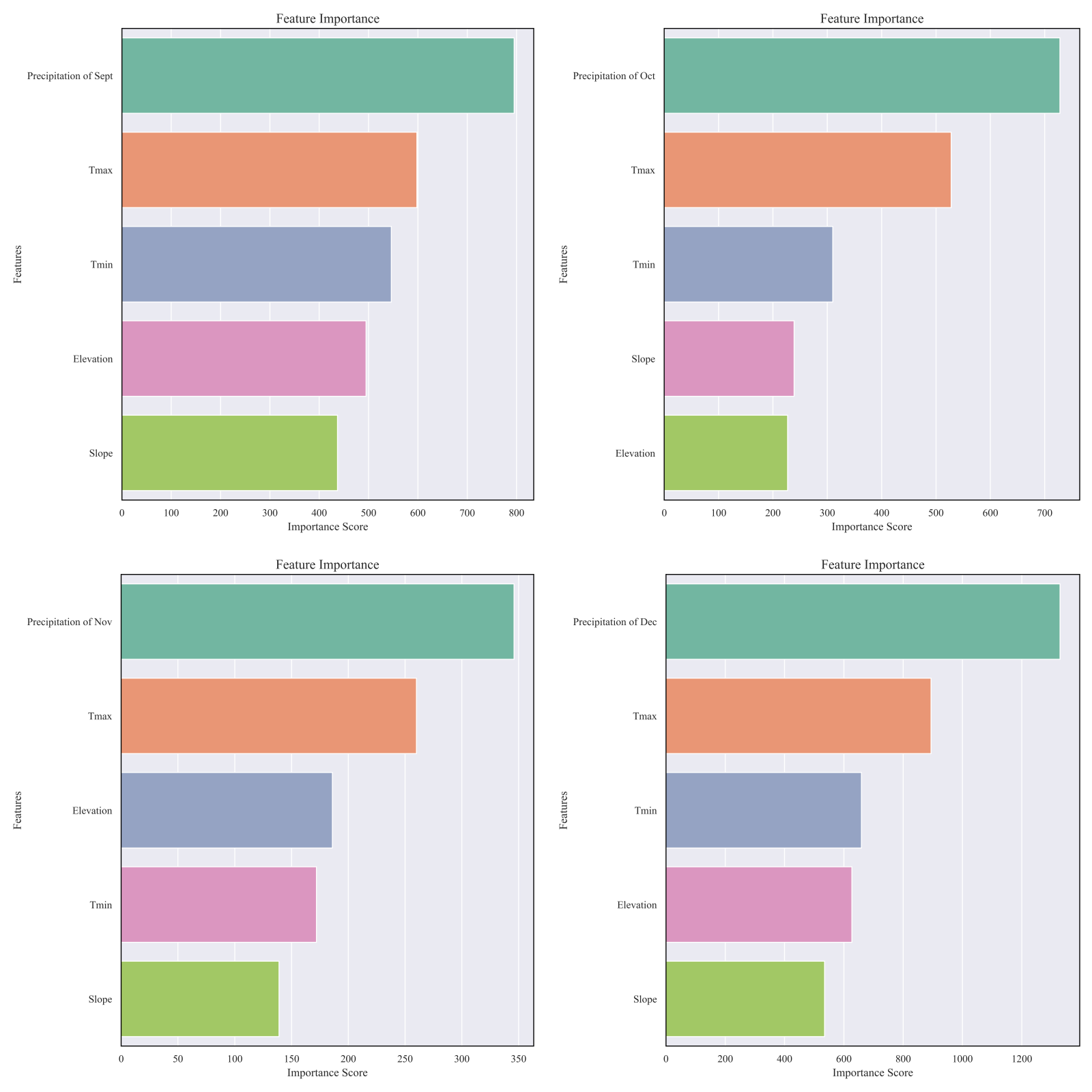


**Supplementary Figure 16.** Feature importance plot for the monthly XGBoost model from September to December. The plots were generated using R version 4.4.3 with the package ggplot2.


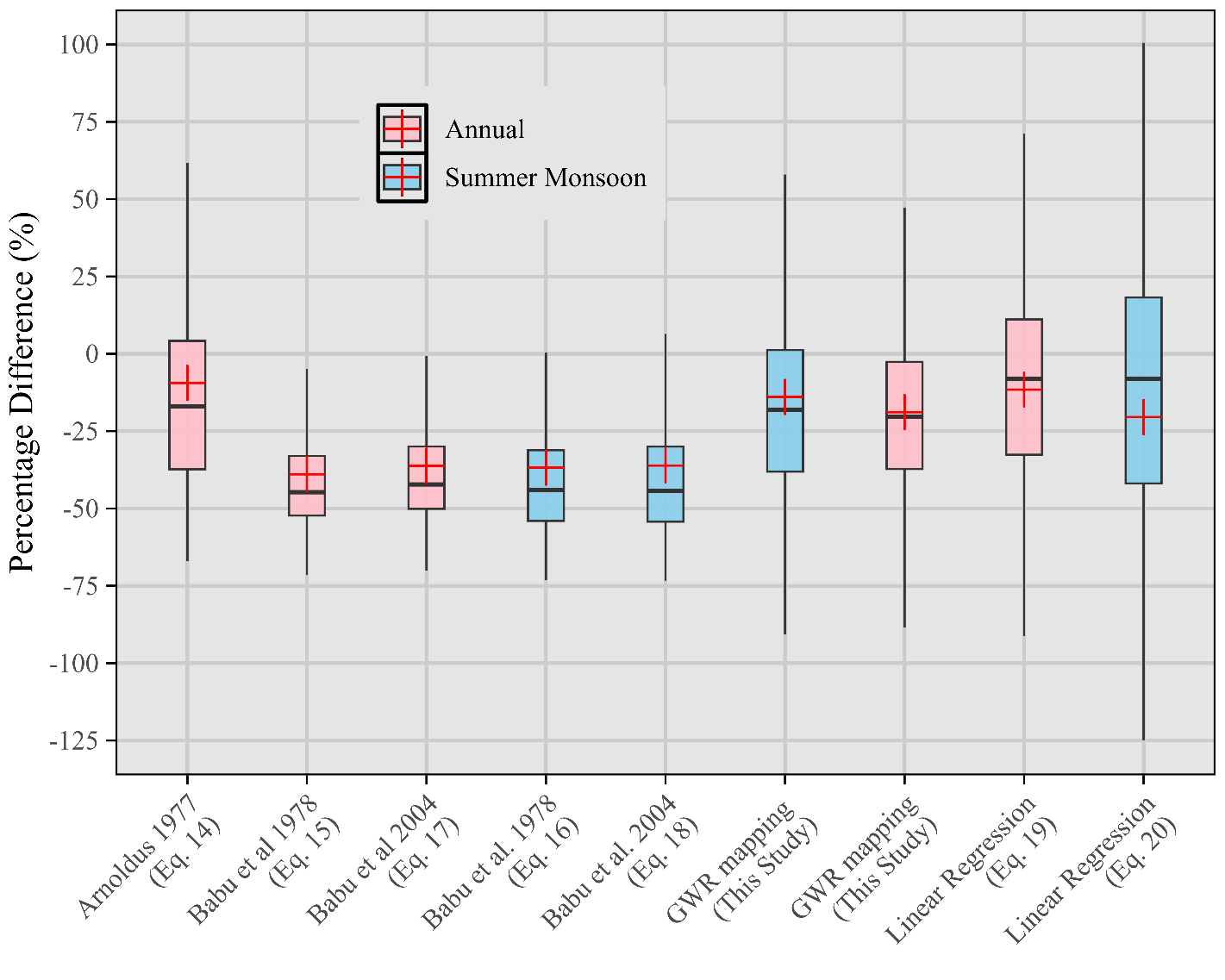


**Supplementary Figure 17.** Percentage error in the different rainfall erosivity existing model. The plots were generated using R version 4.4.3 with the package ggplot2.


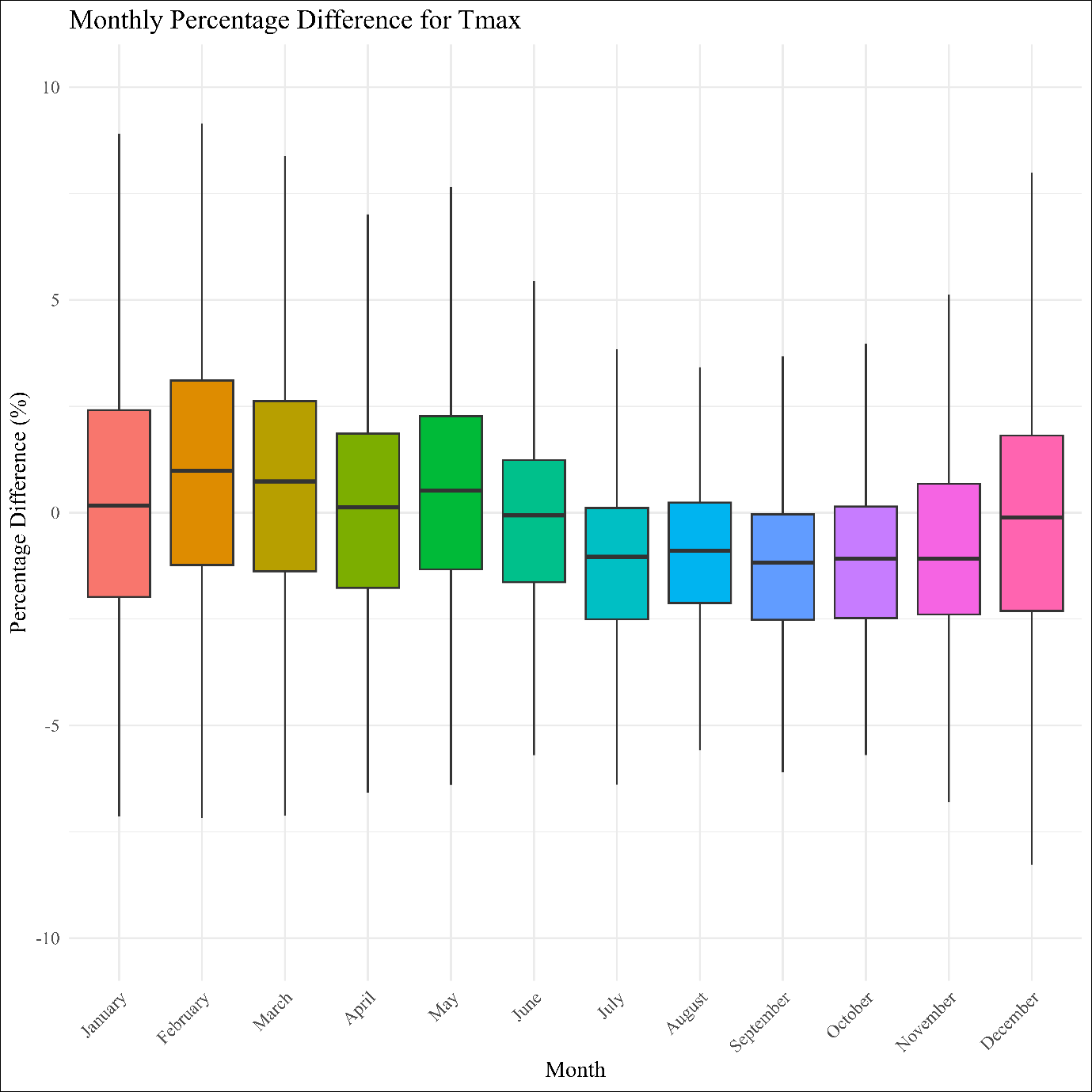


**Supplementary Figure 18.** Percentage error (%) in our adopted methodology for filling missing temperature values from WorldClim, compared to the observed values at the same 510 stations for maximum temperature. The central line of the box plots indicates the median, the edges of the box represent the interquartile range, and the whiskers show the range within 1.5 times the interquartile range. The plots were generated using R version 4.4.3 with the package ggplot2.

**
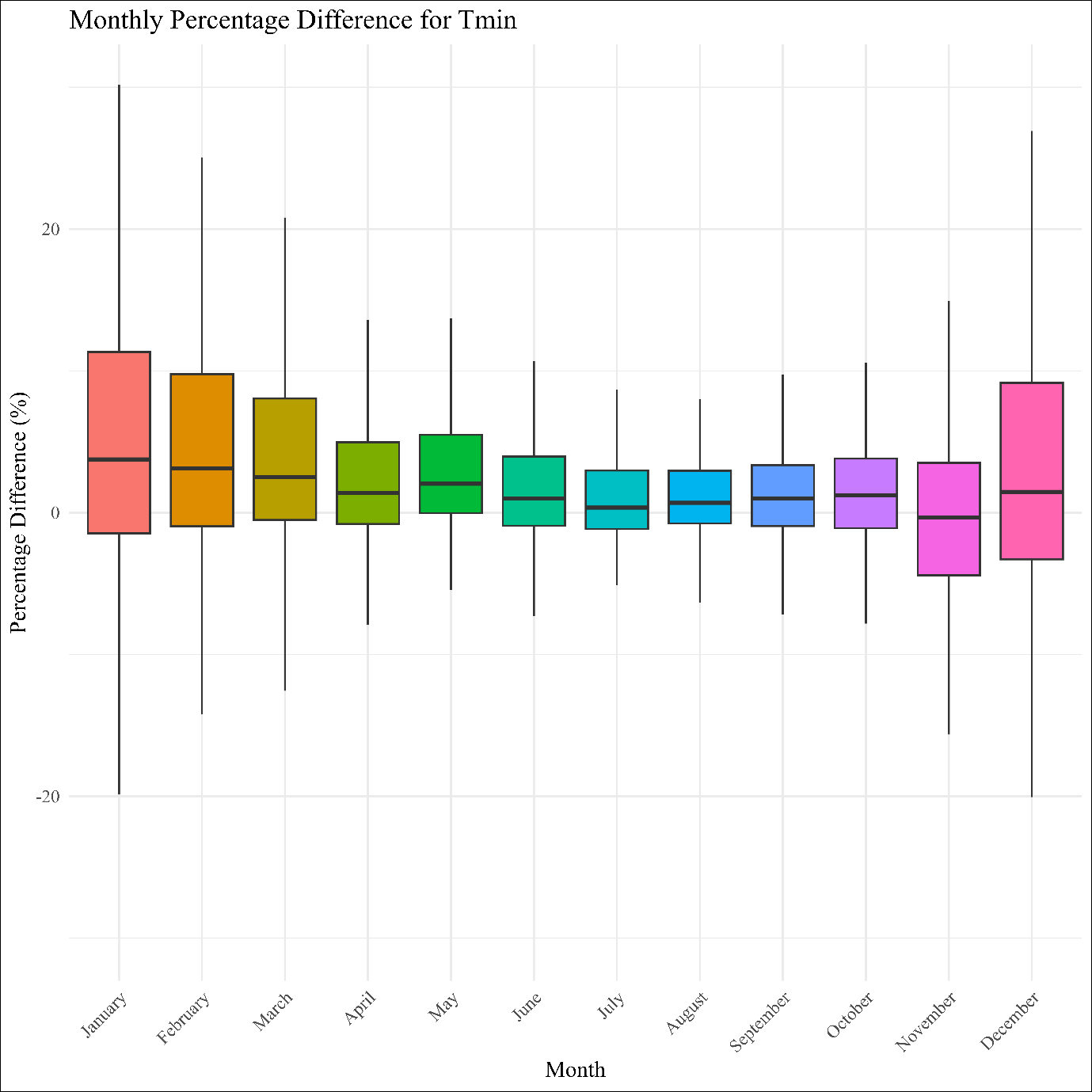
**

**Supplementary Figure 19.** Percentage error (%) in our adopted methodology for filling missing temperature values from WorldClim, compared to the observed values at the same 510 stations for minimum temperature. The central line of the box plots indicates the median, the edges of the box represent the interquartile range, and the whiskers show the range within 1.5 times the interquartile range. The plots were generated using R version 4.4.3 with the package ggplot2.

**Supplementary Table 1.** Hyperparameters (rounded values) for Monthly Rainfall Erosivity Modeling using the XGBoost model.

| **Month** | **nrounds** | **max_depth** | **eta** | **gamma** | **Colsample**  **_bytree** | **min_child**  **_weight** | **subsample** |
| --- | --- | --- | --- | --- | --- | --- | --- |
| January | 180 | 7 | 0.12 | 0.6 | 0.9 | 3 | 0.6 |
| February | 220 | 5 | 0.08 | 1.0 | 0.8 | 2 | 0.9 |
| March | 140 | 6 | 0.15 | 1.4 | 1.0 | 1 | 1.0 |
| April | 260 | 7 | 0.1 | 0.7 | 0.85 | 4 | 0.75 |
| May | 210 | 9 | 0.2 | 1.2 | 1.0 | 3 | 0.6 |
| June | 230 | 6 | 0.05 | 0.3 | 0.8 | 2 | 0.85 |
| July | 270 | 10 | 0.1 | 0.5 | 0.7 | 5 | 0.7 |
| August | 190 | 7 | 0.1 | 0.4 | 1.0 | 3 | 0.9 |
| September | 250 | 6 | 0.07 | 0.2 | 0.85 | 4 | 1.0 |
| October | 160 | 5 | 0.1 | 0.3 | 0.75 | 1 | 0.8 |
| November | 180 | 6 | 0.1 | 0.4 | 1.0 | 2 | 0.7 |
| December | 200 | 7 | 0.1 | 0.6 | 0.8 | 2 | 0.63 |

**Supplementary Table 2.** Estimated maximum and minimum rainfall erosivity and attributes with hourly rainfall data.

| **Month** | **Total erosivity** | **Total kinetic energy** | **Total erosive rainfall** | **Maximum 60-min intensity** | **Total rainfall** | **Number of erosive events** | **Percentage of erosive rainfall to total rainfall** | **Monthly erosivity**  **density** |
| --- | --- | --- | --- | --- | --- | --- | --- | --- |
| Maximum | | | | | | | | |
| January | 439 | 15 | 82 | 28 | 100 | 15 | 100 | 16 |
| February | 4007 | 27 | 147 | 53 | 169 | 8 | 100 | 32 |
| March | 2434.8 | 31 | 158 | 66 | 165 | 12 | 100 | 22 |
| April | 8273 | 90 | 390 | 56 | 436 | 16 | 94 | 23 |
| May | 20756 | 201 | 843 | 64 | 884 | 22 | 96 | 23 |
| June | 48536 | 474 | 2146 | 80 | 2163 | 26 | 99 | 34 |
| July | 60860 | 597 | 2840 | 69 | 2858 | 31 | 99 | 21 |
| August | 24928 | 394 | 2023 | 58 | 2071 | 28 | 98 | 27 |
| September | 17799 | 189 | 882 | 54 | 923 | 22 | 96 | 19 |
| October | 6683 | 81 | 365 | 48 | 391 | 16 | 99 | 25 |
| November | 4893 | 81 | 399 | 42 | 444 | 14 | 100 | 21 |
| December | 2910 | 51 | 247 | 57 | 263 | 9 | 100 | 31 |
| Minimum | | | | | | | | |
| January | 0 | 0 | 0 | 0 | 0 | 0 | 0 | 0 |
| February | 0 | 0 | 0 | 0 | 0 | 0 | 0 | 0 |
| March | 0 | 0 | 0 | 0 | 0 | 0 | 0 | 0 |
| April | 0 | 0 | 0 | 0 | 0 | 0 | 0 | 0 |
| May | 0 | 0 | 0 | 0 | 2 | 0 | 0 | 0 |
| June | 0 | 0 | 0 | 0 | 3 | 0 | 0 | 0 |
| July | 23 | 1 | 2 | 7 | 5 | 1 | 15 | 0 |
| August | 0 | 0 | 0 | 0 | 6 | 0 | 0 | 0 |
| September | 0 | 0 | 0 | 0 | 18 | 0 | 0 | 0 |
| October | 0 | 0 | 0 | 0 | 0 | 0 | 0 | 0 |
| November | 0 | 0 | 0 | 0 | 0 | 0 | 0 | 0 |
| December | 0 | 0 | 0 | 0 | 0 | 0 | 0 | 0 |

**Supplementary Table 3.** Spatial interpolation error metrics for the monthly erosivity mapping using Kriging. PE: Percentage Error (%), R^2^: Coefficient of determination, and RMSE: Root Mean Squared Error (MJ mm ha^‑1^ h^‑1^ month^-1^).

| **Months** | **Median PE** | **RMSE** | **R^2^** |
| --- | --- | --- | --- |
| January | +1.20 | 41 | 0.44 |
| February | -7.16 | 80 | 0.31 |
| March | -12.48 | 83 | 0.48 |
| April | -5.16 | 228 | 0.55 |
| May | -6.32 | 582 | 0.53 |
| June | -10.65 | 1531 | 0.58 |
| July | -11.31 | 2216 | 0.50 |
| August | -8.89 | 1413 | 0.50 |
| September | -5.55 | 766 | 0.45 |
| October | -10.87 | 458 | 0.67 |
| November | -8.76 | 338 | 0.78 |
| December | -2.11 | 138 | 0.76 |

**Supplementary Table 4.** Estimated long-term (1969 – 2021) mean seasonal erosivity (MJ mm ha^-1^ h^‑1^ season^-1^), and annual erosivity (MJ mm ha^-1^ h^-1^ year^-1^) for the five major Köppen-Geiger climate groups ^1^, derived from highly resolved 30-second maps.

| **Season or Annual** | **Tropical** | **Arid** | **Temperate** | **Cold** | **Polar** |
| --- | --- | --- | --- | --- | --- |
| Winter | 113 | 39 | 73 | 214 | 48 |
| Pre-monsoon | 595 | 138 | 494 | 352 | 20 |
| Summer-monsoon | 7537 | 2669 | 5855 | 631 | 361 |
| Post-monsoon | 963 | 317 | 268 | 110 | 42 |
| Annual | 9208 | 3164 | 6690 | 1307 | 469 |

**Supplementary Table 5.** Estimated long-term (1969 – 2021) mean monthly erosivity (MJ mm ha^‑1^ h^‑1^ month^-1^) for the five major Köppen-Geiger climate groups ^1^, derived from highly resolved 30-second erosivity maps.

| **Months** | **Tropical** | **Arid** | **Temperate** | **Cold** | **Polar** |
| --- | --- | --- | --- | --- | --- |
| January | 17 | 6 | 19 | 44 | 8 |
| February | 23 | 13 | 37 | 108 | 12 |
| March | 49 | 13 | 55 | 84 | 1 |
| April | 155 | 39 | 111 | 152 | 10 |
| May | 391 | 86 | 327 | 115 | 9 |
| June | 1608 | 383 | 934 | 83 | 20 |
| July | 2371 | 824 | 1927 | 136 | 63 |
| August | 2100 | 814 | 1804 | 208 | 116 |
| September | 1455 | 647 | 1188 | 203 | 162 |
| October | 705 | 250 | 248 | 85 | 40 |
| November | 257 | 67 | 20 | 25 | 2 |
| December | 72 | 19 | 17 | 63 | 27 |

**Supplementary Table 6.** Frequency of variable importance rankings across 12 months based on SHAP analysis. Variables are grouped based on the number of times they appeared in high (top 3), medium (rank 4–6), and low (rank 7 or below) importance in monthly erosivity estimation models.

| **Variable** | **High Importance (Top 3)** | **Medium Importance (Rank 4—6)** | **Low Importance (Rank ≥ 7)** |
| --- | --- | --- | --- |
| Rainfall | 12 | 0 | 0 |
| Surface Runoff | 9 | 3 | 0 |
| Water Vapor Pressure | 7 | 5 | 0 |
| Distance from Coast | 3 | 9 | 0 |
| Elevation | 4 | 6 | 2 |
| Solar Radiation | 0 | 10 | 2 |
| Soil Moisture | 1 | 2 | 9 |
| Wind Speed | 0 | 1 | 11 |
| Soil Type | 0 | 0 | 12 |
| Slope | 0 | 0 | 12 |
| Land Use Type | 0 | 0 | 12 |

**Supplementary Table 7.** Modified Mann-Kendall Trend Test p-values and FDR-adjusted p-values for Monthly Rainfall Erosivity and Associated Variables.

| **Month** | **Total erosivity** | **Total kinetic energy** | **Total erosive rainfall** | **Maximum 60-min intensity** | **Total rainfall** | **Number of erosive events** | **Percentage of erosive rainfall to total rainfall** | **Monthly erosivity**  **density** |
| --- | --- | --- | --- | --- | --- | --- | --- | --- |
| Modified M-K p-values | | | | | | | | |
| January | **0.005** | **0.026** | **0.032** | **0.001** | 0.076 | 0.345 | **0.003** | **0.001** |
| February | 0.523 | 0.508 | 0.641 | 0.160 | 0.801 | 0.431 | 0.092 | 0.147 |
| March | **0.006** | **0.025** | **0.032** | 0.300 | 0.085 | 0.060 | **0.022** | 0.200 |
| April | 0.485 | 0.072 | 0.148 | 0.868 | 0.151 | 0.175 | 0.125 | 0.890 |
| May | 0.241 | 0.386 | 0.629 | **0.005** | 0.565 | 0.684 | 0.274 | **0.035** |
| June | 1.00 | 0.921 | 0.993 | 0.429 | 0.982 | 0.933 | 0.980 | 0.742 |
| July | 0.358 | 0.088 | **0.024** | 0.197 | 0.085 | 0.618 | **0.023** | 0.300 |
| August | 0.929 | 0.429 | 0.200 | 0.327 | 0.211 | **0.047** | 0.457 | 0.082 |
| September | 0.378 | 0.293 | 0.259 | 0.181 | 0.330 | 0.788 | **0.014** | 0.253 |
| October | **0.010** | **0.008** | **0.015** | **0.000** | **0.021** | **0.024** | **0.020** | **0.001** |
| November | 0.177 | 0.073 | 0.066 | **0.000** | 0.055 | **0.000** | **0.020** | 0.390 |
| December | **0.009** | **0.014** | **0.014** | 0.099 | **0.020** | 0.931 | **0.032** | **0.001** |
| Adjusted p-values | | | | | | | | |
| January | **0.047** | 0.088 | 0.100 | **0.014** | 0.188 | 0.495 | **0.032** | **0.012** |
| February | 0.643 | 0.633 | 0.751 | 0.308 | 0.894 | 0.560 | 0.200 | 0.295 |
| March | 0.052 | 0.088 | 0.100 | 0.450 | 0.194 | 0.163 | 0.088 | 0.343 |
| April | 0.613 | 0.185 | 0.295 | 0.958 | 0.296 | 0.327 | 0.260 | 0.971 |
| May | 0.398 | 0.527 | 0.745 | **0.047** | 0.687 | 0.792 | 0.431 | 0.105 |
| June | 1.000 | 0.973 | 1.000 | 0.559 | 1.000 | 0.973 | 1.000 | 0.847 |
| July | 0.506 | 0.196 | 0.088 | 0.343 | 0.194 | 0.742 | 0.088 | 0.451 |
| August | 0.973 | 0.559 | 0.343 | 0.480 | 0.356 | 0.137 | 0.585 | 0.194 |
| September | 0.526 | 0.451 | 0.415 | 0.329 | 0.478 | 0.890 | 0.080 | 0.412 |
| October | 0.068 | 0.062 | 0.080 | **0.010** | 0.088 | 0.088 | 0.088 | **0.012** |
| November | 0.327 | 0.185 | 0.176 | **0.000** | 0.156 | **0.000** | 0.088 | 0.527 |
| December | 0.068 | 0.080 | 0.080 | 0.211 | 0.088 | 0.973 | 0.100 | **0.018** |

**Supplementary Table 8.** Comparison of correction factors used to adjust rainfall erosivity estimates derived from hourly rainfall data instead of high-resolution breakpoint data, as reported in various studies across the globe. The correction factor adopted in this study is compared to those references. The percentage difference is calculated using the formula:

Percentage difference = ($\frac{Correction factor used in this study - Reference correction factor}{Reference correction factor}) \times100$

| **Study Area** | **Referenced correction factor** | **Reference** | **Percentage error (%)** |
| --- | --- | --- | --- |
| China | 1.871 | Yue et al ^2^ | +6.89 |
| Southeastern  Georgia, US | 1.837 | Williams and Sheridan ^3^ | +8.87 |
| Europe | 2.081 | Panagos et al.^4^ | -3.89 |
| China | 1.73 | Yin et al.^5^ | +15.61 |
| Germany | 2.05 | Fischer et al.^6^ | -2.44 |
| US | Range (1.08 to 3.16) × 1.0667 | Renard et al.^7^ | +73.61 to -40.67 |

**Supplementary Table 9.** ANOVA results showing the p-values and F-values for the effects of rainfall category, elevation, land use, and soil type on the residual of the rainfall erosivity estimated across different months. Significant factors (p-value < 0.05) are highlighted.

| **Month** | **Rainfall Category** | | **Elevation** | | **Land Use** | | **Soil Type** | |
| --- | --- | --- | --- | --- | --- | --- | --- | --- |
|  | p-value | F-value | p-value | F-value | p-value | F-value | p-value | F-value |
| January | **0.014** | 4.30 | **0.006** | 5.190 | **0.006** | 3.018 | 0.444 | 1.005 |
| February | 0.315 | 1.16 | **0.026** | 3.64 | 0.109 | 1.74 | 0.605 | 0.852 |
| March | 0.091 | 2.40 | 0.122 | 2.11 | **0.003** | 3.28 | 0.811 | 0.65 |
| April | 0.271 | 1.31 | **0.004** | 5.57 | **<0.000**  **001** | 6.96 | 0.505 | 0.94 |
| May | 0.153 | 1.88 | **0.025** | 3.70 | 0.744 | 0.58 | 0.800 | 0.66 |
| June | 0.438 | 0.83 | **0.040** | 3.21 | 0.541 | 0.84 | 0.902 | 0.54 |
| July | 0.168 | 1.78 | 0.065 | 2.74 | 0.239 | 1.33 | **<0.00**  **0001** | 8.17 |
| August | **0.011** | 4.55 | 0.205 | 1.59 | 0.627 | 0.73 | **0.000**  **0001** | 5.91 |
| September | 0.051 | 2.97 | 0.266 | 1.32 | 0.238 | 1.37 | **0.038** | 1.79 |
| October | 0.114 | 2.18 | 0.105 | 2.26 | 0.723 | 0.61 | 0.981 | 0.36 |
| November | **0.001** | 6.96 | 0.450 | 0.80 | **0.030** | 2.33 | 0.195 | 1.32 |
| December | **0.016** | 4.15 | **<0.001** | 13.52 | **<0.000**  **001** | 5.60 | 0.452 | 1.00 |

References:

1. Beck, H. E. *et al.* Present and future köppen-geiger climate classification maps at 1-km resolution. *Sci. Data* **5**, 1–12 (2018).

2. Yue, T. *et al.* Effect of time resolution of rainfall measurements on the erosivity factor in the USLE in China. *Int. Soil Water Conserv. Res.* **8**, 373–382 (2020).

3. Williams, R. G. & Sheridan, J. M. Effect of rainfall measurement time and depth resolution on EI calculation. *Trans. Am. Soc. Agric. Eng.* **34**, 402–406 (1991).

4. Panagos, P. *et al.* Monthly rainfall erosivity: Conversion factors for different time resolutions and regional assessments. *Water Switz.* **8**, 119 (2016).

5. Yin, S., Xie, Y., Nearing, M. A. & Wang, C. Estimation of rainfall erosivity using 5- to 60-minute fixed-interval rainfall data from China. *Catena* **70**, 306–312 (2007).

6. Fischer, F. K., Winterrath, T. & Auerswald, K. Temporal- and spatial-scale and positional effects on rain erosivity derived from point-scale and contiguous rain data. *Hydrol. Earth Syst. Sci.* **22**, 6505–6518 (2018).

7. Renard, K., Foster, G., Weesies, G., McCool, D. & Yoder, D. Predicting soil erosion by water: a guide to conservation planning with the Revised Universal Soil Loss Equation (RUSLE). *Agric. Handb. No 703* 404 (1997).
